# Specific T-cell subsets have a role in anti-viral immunity and pathogenesis but not viral dynamics or onwards vector transmission of an important livestock arbovirus

**DOI:** 10.3389/fimmu.2024.1328820

**Published:** 2024-01-31

**Authors:** Kerry Newbrook, Nakibul Khan, Aimee Fisher, Karen Chong, Simon Gubbins, William C. Davies, Christopher Sanders, Marc Guimerà Busquets, Lyndsay Cooke, Amanda Corla, Martin Ashby, John Flannery, Carrie Batten, Jessica E. Stokes, Beatriz Sanz-Bernardo, Simon Carpenter, Katy Moffat, Karin E. Darpel

**Affiliations:** ^1^ Orbivirus Research, The Pirbright Institute, Woking, United Kingdom; ^2^ Department of Biology, University of York, York, United Kingdom; ^3^ School of Biosciences AND School of Veterinary Medicine, University of Surrey, Guildford, United Kingdom; ^4^ Transmission Biology, The Pirbright Institute, Woking, United Kingdom; ^5^ Department of Veterinary Microbiology and Pathology, Washington State University, Pullman, WA, United States; ^6^ Entomology, The Pirbright Institute, Woking, United Kingdom; ^7^ Non Vesicular Reference Laboratory, The Pirbright Institute, Woking, United Kingdom; ^8^ Large Deoxyribonucleic Acid (DNA), Viruses, The Pirbright Institute, Woking, United Kingdom; ^9^ Flow Cytometry, The Pirbright Institute, Woking, United Kingdom; ^10^ Department of Diagnostics and Development, Institute of Virology and Immunology, Mittelhäusern, Switzerland; ^11^ Department of Infectious Diseases and Pathobiology, Vetsuisse Faculty, University of Bern, Bern, Switzerland

**Keywords:** T cell, Bluetongue virus, *Culicoides*, *Orbivirus*, pathogenesis, immunity

## Abstract

**Introduction:**

Bluetongue virus (BTV) is an arthropod-borne *Orbivirus* that is almost solely transmitted by *Culicoides* biting midges and causes a globally important haemorrhagic disease, bluetongue (BT), in susceptible ruminants. Infection with BTV is characterised by immunosuppression and substantial lymphopenia at peak viraemia in the host.

**Methods:**

In this study, the role of cell-mediated immunity and specific T-cell subsets in BTV pathogenesis, clinical outcome, viral dynamics, immune protection, and onwards transmission to a susceptible *Culicoides* vector is defined in unprecedented detail for the first time, using an *in vivo* arboviral infection model system that closely mirrors natural infection and transmission of BTV. Individual circulating CD4^+^, CD8^+^, or WC1^+^ γδ T-cell subsets in sheep were depleted through the administration of specific monoclonal antibodies.

**Results:**

The absence of cytotoxic CD8^+^ T cells was consistently associated with less severe clinical signs of BT, whilst the absence of CD4^+^ and WC1^+^ γδ T cells both resulted in an increased clinical severity. The absence of CD4^+^ T cells also impaired both a timely protective neutralising antibody response and the production of IgG antibodies targeting BTV non-structural protein, NS2, highlighting that the CD4^+^ T-cell subset is important for a timely protective immune response. T cells did not influence viral replication characteristics, including onset/dynamics of viraemia, shedding, or onwards transmission of BTV to *Culicoides*. We also highlight differences in T-cell dependency for the generation of immunoglobulin subclasses targeting BTV NS2 and the structural protein, VP7.

**Discussion:**

This study identifies a diverse repertoire of T-cell functions during BTV infection in sheep, particularly in inducing specific anti-viral immune responses and disease manifestation, and will support more effective vaccination strategies.

## 1 Introduction

Bluetongue (BT) is an infectious, non-contagious, haemorrhagic disease of both domestic and wild ruminants, which is associated with particularly severe clinical disease in sheep. Clinical signs typically include facial oedema, breathing difficulties, conjunctivitis, fever, haemorrhages, coronitis, and lameness (1). The causative agent of BT is the arthropod-borne pathogen, bluetongue virus (BTV), which is biologically transmitted between its mammalian hosts by susceptible *Culicoides* biting midges of the Family Ceratopogonidae (2). BTV is the type species of the *Orbivirus* genus (Family: *Sedoreoviridae*) and is comprised of 10 segments of double-stranded RNA, encoding seven structural (VP1–7) and at least four non-structural (NS1–NS4) proteins. There are currently at least 29 recognised BTV serotypes (3). Over the last two decades, there have been multiple widespread incursions of BTV across large parts of Northern Europe (4, 5), contributing to its substantial global economic burden (6–8). As a significant and continuing global threat to livestock production and food security, BT is notifiable to the World Organisation for Animal Health.

Humoral immunity is thought to be a key driver of protection from BTV infection in ruminants. Neutralising antibodies, raised predominantly against the BTV outer capsid protein, VP2, provide protection against re-infection with strains of the homologous serotype (9–11). Short-lived, partial protection against strains from heterologous BTV serotypes has also been demonstrated (12, 13), but often in the absence of neutralising antibodies (14–16), thereby indicating additional mechanisms at play. T cells have been a major target of study in innate and adaptive immune responses to BTV infection (17, 18), particularly when exploring cross-serotype immune protection. CD8^+^ cytotoxic T cells have demonstrated cross-reactivity against heterologous BTV serotypes (19, 20) and confer some partial cross-serotype protection against BTV in sheep (14, 21, 22). Furthermore, CD4^+^ and CD8^+^ T cells have both been shown to recognise epitopes on structural (VP2 and VP7) and non-structural BTV proteins (NS1) (19, 23–26).

BTV infection of sheep is characterised by acute immunosuppression, which is thought to facilitate its characteristic prolonged viraemia through evasion of the host immune response (27). Specific changes in T-cell dynamics have been identified, including a pan-lymphopenia at peak viraemia followed by a transient increase in the CD8^+^ T-cell population (17, 28, 29). It is also known that ovine CD4^+^ and WC1^+^ γδ T cells and bovine CD4^+^, CD8^+^, and WC1^+^ γδ T cells can all become productively infected with BTV (30–34). T-cell depletion has been achieved in large ruminants by administration of specific monoclonal antibodies (mAbs) to “knock out” circulating T cells via antibody-mediated, complement-dependent cytotoxicity. This method has been used to investigate T-cell function in cattle, goats, and pigs during infection with foot-and-mouth disease virus, peste-des-petits ruminants virus, and African swine fever virus (35–37).

Here, for the first time, we use mAb-mediated T-cell depletion to investigate the function of specific T-cell subsets (CD4^+^, CD8^+^, and WC1^+^ γδ) in clinical disease outcomes, viral dynamics, humoral and cellular immune responses, and onwards vector transmission during BTV infection in sheep. The use of a natural host–virus–vector transmission model enabled the investigation of pathogenesis and anti-viral immunity *in vivo* in unprecedented detail. We demonstrate that individual T-cell subsets modulate clinical outcome, with the absence of CD8^+^ T cells associated with lower clinical scores and higher survival, and loss of CD4^+^ and WC1^+^ γδ T cells resulting in higher clinical scores and severity. The loss of CD4^+^ T cells appeared to impair a timely protective, neutralising antibody response and the production of IgG antibodies targeting non-structural protein, NS2, highlighting the likely importance of this T-cell subset. We also highlight that the induction of specific immunoglobulin classes recognising either VP7 or NS2 antigen differed in their T-cell dependency. T cells were not, however, found to influence the onset or dynamics of BTV viraemia, viral shedding, or onwards transmission to a susceptible *Culicoides* vector.

## 2 Materials and methods

### 2.1 Cell lines

KC cells, derived from 2-day-old *Culicoides sonorensis* embryos (38), and BSR cells, a clone of the baby hamster kidney (BHK)-21 cell line, were maintained as previously described (39). Vero cells, obtained from the European Collection of Authenticated Cell Cultures (Porton Down, UK), were maintained as for BSR cells, except culture media contained 10% (v/v) foetal bovine serum (Thermo Fisher Scientific, Loughborough, UK).

### 2.2 Insects

Mixed-sex, newly emerged adult *C. sonorensis* (PIRB-S-3 strain) individuals (40), obtained from the AA colony line at The Pirbright Institute (41), were used in all *in vivo* experiments. Insects were provided in netted cardboard pots (Watkins and Doncaster, Leominster, UK) of ~400 individuals, maintained daily with 10% (w/v) sucrose solution (provided via cotton wool) at 25°C under 70%–90% humidity.

### 2.3 Virus propagation

BTV-4 MOR2009/07 [KC1], a strain of moderate clinical severity with a high infection rate of (AA) *C. sonorensis* (42), was obtained from the *Orbivirus* Reference Collection (The Pirbright Institute). Serial passage on KC cells (7 days, 28°C) generated BTV-4 MOR2009/07 [KC2] infectious tissue culture supernatant (TCS) for *in vivo* studies. Harvest, storage, and quantification (end-point titration to determine 50% tissue culture infective dose (TCID_50_)) of the TCS was performed as previously described (39, 42), except that KC cells were incubated at 28°C, with secondary antibody diluted at 1:250. Serotype specificity was confirmed by qRT-PCR targeting BTV segment 2 (43). BTV-4 MOR2009/07 [KC1] was serially passaged four times in BSR cells to generate BTV-4 MOR2009/07 [KC1, BSR4] for the serum neutralisation test (SNT).

### 2.4 BTV infection of *C. sonorensis*

*C. sonorensis* were fed a mixed suspension of defibrinated horse blood (TCS Biosciences Ltd., Buckingham, UK) and BTV-4 MOR2009/07 [KC2] (3:1 blood:virus ratio, 3 ml) through a parafilm membrane using the Hemotek system (Hemotek Ltd., Blackburn, UK). Final blood meal virus titres for infection were between 6.57 and 7.57 log_10_ TCID_50_/ml. Fully engorged, blood-fed individuals were collected into netted cardboard pots (80 individuals per pot) by controlled CO_2_ anaesthesia using a light stereomicroscope (Leica Microsystems (UK) Ltd., Milton Keynes, UK) and maintained for 7 or 8 days as above prior to feeding on sheep.

### 2.5 *In vivo* depletion studies

Thirty-three 12-month-old female British mule (Texel terminal sire) sheep were used (same farm/breeding lineage) for all *in vivo* depletion experiments. All sheep tested negative for serum anti-BTV VP7 antibodies using the ID Screen^®^ bluetongue competitive ELISA (cELISA) (Innovative Diagnostics, Grabels, France) and for BTV RNA in ethylenediaminetetraacetic acid (EDTA) blood by qRT-PCR (44) prior to the experiment. The study was performed at The Pirbright Institute’s high containment animal facility as five separate experiments, with five or seven animals per replicate. Sheep were randomly assigned to each depletion group but stratified to ensure representatives of each depletion group in each experimental replicate, except experiment 1, which had no CD4^+^ T cell-depleted sheep. Sheep were housed in groups and fed hay and water *ad libitum* and supplemented with grain pellets. Sheep were weighed prior to the experiment (range 39–47 kg).

Sheep were administered daily intravenous injections of specific mAbs (**Table 1**) for 5 days (−2 to 2 days post infection (dpi)) to deplete either CD4^+^ (n = 7), CD8^+^ (n = 7), or WC1^+^ γδ (n = 7) T cells. A further seven sheep were inoculated with an isotype-matched mAb targeting Turkey rhinotracheitis virus (TRTV) as a mock-depleted control. Each sheep received a total final dose of 2 mg/kg mAb, gradually increasing dosage to minimise adverse effects: 0.75 mg in the morning and then 1.75 mg in the evening at −2 dpi, 15 mg at −1 dpi, and the remaining dosage titrated in over 0 dpi to 2 dpi based on body weight. Finadyne^®^ solution (National Veterinary Services, Camberley, UK) was intravenously administered to all sheep prior to the first three to four mAb injections to minimise adverse reactions of cell lysis (2.2 mg/kg total dose). Mild adverse effects (e.g., increased breathing rate, slowness, and short-lived general depression) were observed for 1–2 hours at −2 dpi and −1 dpi, primarily in CD8^+^ T cell-depleted sheep, coinciding with the greatest decrease in T-cell populations.

**Table 1:** Monoclonal antibodies used to deplete or mock-deplete specific T-cell subsets.

Antibody target	Clone	Isotype	Source	References
CD4	GC1a	IgG2a	Washington University Monoclonal Antibody Centre	(45)
CD8	CC63	IgG2a	Antibody production services	(46)
WC1	CC15	IgG2a	Antibody production services	(47)
TRTV (control)	TRT3	IgG2a	Antibody production services	(48)

**Figure 1:**
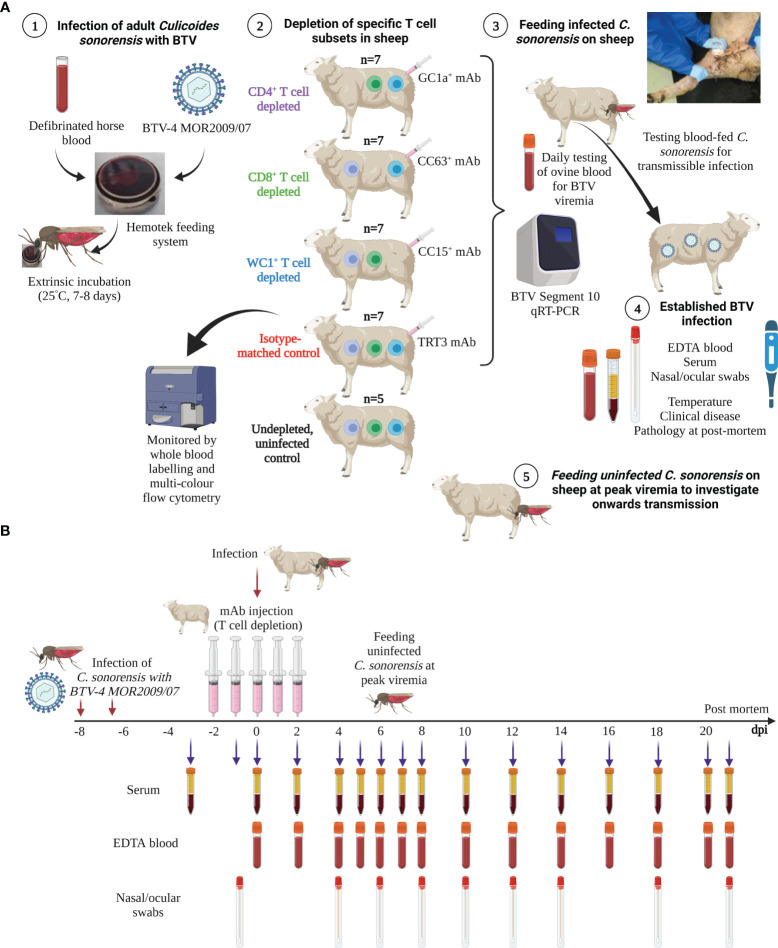
Schematic diagram of experimental design and timeline. **(A)** Adult *Culicoides sonorensis* were fed on a defibrinated horse blood–virus mixture using the Hemotek feeding system to enable uptake of BTV-4 MOR2009/07. C*. sonorensis* were incubated at 25°C for 7–8 days (extrinsic incubation period) to enable virus dissemination to the salivary gland. Midges were subsequently allowed to blood-feed on sheep depleted of CD4^+^ (n = 7), CD8^+^ (n = 7), or WC1^+^ γδ T cells, which was achieved by administration of specific monoclonal antibodies (mAbs; GC1a, CC63, CC15), or on mock-depleted sheep (n = 7), achieved using an isotype-matched mAb raised against Turkey rhinotracheitis virus (TRT3). One sheep within each experimental replicate (n = 5) was not depleted and not infected (negative control) to monitor for potential contact transmission. Following blood-feeding, engorged *C*. *sonorensis* females were collected to determine the numbers of transmissibly infected (TI) individuals that had taken a blood meal by RNA extraction and bluetongue virus (BTV) (segment 10-specific) qRT-PCR. Whole blood was taken daily or every other day for immunophenotyping, using specific T- and B-cell mAbs, to monitor the T-cell depletion and specific immune cell population dynamics during infection. Whole (ethylenediaminetetraacetic acid (EDTA) and heparin) blood, clotted blood (for serum), and nasal and ocular swabs were taken from all sheep at various time points during infection (see panel **B**) to monitor viraemia dynamics, host antibody responses, and viral shedding. Temperatures and clinical disease scoring were recorded daily. At peak viraemia, uninfected adult *C*. *sonorensis* were allowed to blood-feed on each sheep, and engorged females were collected immediately (day 0) to obtain a virus uptake baseline or following extrinsic incubation (day 8) to monitor infection rate and therefore onwards transmission rate to the *Culicoides* vector. **(B)** Timeline of BTV infection, T-cell depletion, and sample collection for the representative *in vivo* BTV transmission study. Schematic images created in BioRender.com.

BTV infection was established in sheep using our natural *in vivo* host–virus–vector transmission model (**Figure 1A**). At 0 dpi, two pots of (potentially) infected *C. sonorensis* were continuously held on the skin (inner thigh) of each sheep for 10 minutes to allow insect blood-feeding. One sheep in each experimental replicate (n = 5) remained uninfected (and un-depleted) to monitor for contact transmission (**Figure 1A**). The infection rates of these blood-fed *C. sonorensis* individuals were then determined as previously described (42). Uninfected *C. sonorensis* (two pots per sheep) were blood-fed on each sheep at peak viraemia (as above) to investigate the BTV infection rate as an indicator for onwards transmission to a susceptible *Culicoides* vector. Between eight and 16 fully engorged, blood-fed individuals were collected from each sheep under controlled CO_2_ anaesthesia and immediately processed (as previously described (42)) to determine the initial quantity of BTV imbibed by *C. sonorensis* specifically from each sheep (D0 midges). Then, 150 fully engorged, blood-fed *Culicoides* per sheep were further collected into netted cardboard pots (Watkins and Doncaster) and maintained for 8 days, as above, to complete the extrinsic incubation period. Surviving individuals were processed for RNA extraction/qRT-PCR as above, and replicating virus was inferred by comparison with baseline values from D0 midges fed on the same sheep.

Whole blood was sampled from the jugular vein of each sheep into vacutainers containing EDTA, heparin, and no anti-coagulant (clotted) at −3/−2/−1 dpi, 0 dpi, 2 dpi, 4 dpi, 5 dpi, 6 dpi, 7 dpi, 8 dpi, 10 dpi, 12 dpi, 14 dpi, 16 dpi, 18 dpi, and 20–22 dpi (**Figure 1B**). Whole EDTA blood was stored at 4°C. Clotted blood was centrifuged (1,000 × *g*, 10 minutes, room temperature), and serum was collected immediately for storage (−80°C). Nasal and ocular cavities were sampled using sterile Deltalab plain (PS+Viscose) DNA swabs (Medline Scientific Ltd., Oxon, UK) at −1 dpi, 4 dpi, 6 dpi, 8 dpi, 10 dpi, 12 dpi, 14 dpi, 18 dpi, and 20–22 dpi (**Figure 1B**) and stored (4°C) in 1 ml Dulbecco’s phosphate-buffered saline (DPBS; Thermo Fisher Scientific). Sheep were euthanised by intravenous anaesthesia overdose (pentobarbital sodium, 140 mg/kg) at the end of the study (20–22 dpi) or immediately upon reaching any or a combination of pre-defined clinical thresholds (clinical humane endpoints). All sheep were assessed daily against these endpoints, which included persistent pyrexia/coughing, sustained (nasal, oral, and conjunctival) discharge, continued/haemorrhagic diarrhoea, behavioural changes, food intake, respiratory distress, and lameness. Pathological examinations were performed post-mortem to exclude any pre-existing underlying health conditions and confirm BTV-associated pathology.

### 2.6 Clinical scoring

Rectal temperatures and clinical signs were recorded daily for each sheep using pre-defined clinical criteria standardised across all experiments. Clinical signs were scored by at least two different observers in each experiment (three animal technicians and one investigator in total) to minimise observer bias. Clinical observers were not blinded to treatment groups due to the complex study design and the danger of incorrect mAb administration. Clinical signs were scored in 0.5 increments from 0 to 3 according to increasing severity. A cumulative clinical score was calculated daily for each animal based on fever, food intake, behaviour, redness of eyes/conjunctivitis, redness of oral/nasal mucosal membranes, facial swelling/oedema, salivation, ulcers, nasal discharge, cough, breathing difficulties, and average foot score. Fever score was calculated based on an animal’s baseline rectal temperature (averaged over at least 3 days prior to mAb administration), scoring 1 for ≥1°C above baseline, 2 for ≥1.5°C above baseline, and 3 for ≥2°C above baseline. Food intake was scored as an average of two daily meals: normal food intake (0), reduced hay/concentrate intake (1), only eating hay (no concentrate) (2), or no food intake (3). Behavioural score encompassed apathy/slowness (0.5 to 1), reluctance to get up unaided/prolonged daily group separation (1.5 to 2), or reluctance to get up in response to direct stimulation/body posture indicative of pain (2.5 to 3). Foot score was an average cumulative score of redness, warmness, bleeding, and lameness across all four feet. A severity index was calculated for each day of infection within each treatment group as the sum of all clinical scores for all animals within the group divided by the number of animals alive each day.

### 2.7 Whole-blood immunophenotyping using flow cytometry

Ovine whole blood was immunolabelled using a panel of T- and B-cell markers and analysed by flow cytometry to assess the efficiency of T-cell depletion and dynamics of specific immune cell populations in the blood during BTV infection (**Figure 1B**). T- and B-cell subsets were identified using a second set of lineage marker antibodies of different isotypes to the depletion antibodies to avoid potential uncertainties of epitope masking. Prior *in vitro* assessment of single and dual labelling was conducted to confirm that antibody combinations did not interfere with each other. Whole heparin blood (100 µl), in triplicate, was blocked (30 minutes, room temperature) with 10% (v/v) mouse serum (Sigma Aldrich, Poole, UK) and then labelled with 20 µl mAb cocktail (anti-CD4, anti-CD8, anti-WC1, anti-γδ T-cell receptor, and/or anti-CD21; **Table 2**) with 1 µl Zombie NIR fixable viability stain (BioLegend, San Diego, CA, USA), included enabling exclusion of dead cells during analysis. Each mAb was conjugated to a fluorochrome using a Lightning-Link^®^ Alexa Fluor 647 (Novus Biologicals, Abingdon, UK) or Alexa Fluor™ 488/Pacific Blue (PACB) (Thermo Fisher Scientific) antibody labelling kit, as per manufacturer’s instructions. Conjugates were titrated on ovine whole blood prior to use to determine optimal dilutions. The immunophenotyping panel used in experiment 1 (sheep 1 to 5) included all five mAbs; however, subsequent experiments (sheep 6 to 33) used two separate panels (CD4 and CD21; CD8, WC1, and γδ TcR) to improve the resolution of cell populations. Following labelling, cells were simultaneously fixed and red blood cells lysed by incubation (30 minutes, room temperature) with 2 ml fix/lyse solution (eBioscience™ one-step; Thermo Fisher Scientific). Data were collected using a BD LSR Fortessa flow cytometer with BD FACSDIVA™ software (BD Biosciences, San Jose, CA, USA) and analysed with FCS Express version 6 (*De Novo* Software, Pasadena, CA, USA). Compensation was carried out using blood labelled with individual fluorochrome conjugates (single colours). Data were gated on cells, singlets, and live cells and then into specific subsets based on expression markers (representative gating strategy in **Supplementary Figure S1**).

**Table 2:** Monoclonal antibodies used to immunophenotype ovine whole blood by flow cytometry.

Antibody target	Clone	Antibody source	Fluorochrome conjugate	Reference
CD4	17D	The Pirbright Institute	AF647	(49)
CD21	CC21	Bio-Rad	AF488	(50)
CD8	CC58	Bio-Rad	AF647	(51)
WC1	CC39	The Pirbright Institute	PACB	(52)
γδ TcR	86D	The Pirbright Institute	AF488	(53)

Whilst T-cell populations analysed by flow cytometry are typically reported as a percentage of the total lymphocyte/monocyte population (29, 36, 54), these percentages are directly influenced by changes in other immune cell populations. Here, a specific volume (therefore concentration) of microsphere counting beads (100 µl 123count ebeads™ or 50 µl CountBright™ Absolute; Thermo Fisher Scientific) was added to each blood sample, according to manufacturer’s instructions, and 5,000 bead events were collected to enable calculation of absolute cell numbers per ml blood. Due to high variation between individuals in baseline (pre-depletion) absolute cell numbers for each T/B cell subset, post-depletion absolute cell numbers on each day for each T/B cell subset were normalised to the individual sheep’s calculated baseline (pre-depletion) absolute cell number (average −3 dpi to −2 dpi) for that subset (post-depletion cell number per ml blood/average baseline cell number per ml blood × 100). To provide a representative estimate of absolute cell numbers collected per sample, we averaged cell numbers collected across three technical replicates from one animal of each (n = 5) experimental replicate.

### 2.8 RNA extraction

Total RNA was extracted from 100 µl EDTA blood, *C. sonorensis* homogenates, or pooled swab preparations using an LSI MagVet™ Universal isolation kit and KingFisher Flex automated extraction robot (Thermo Fisher Scientific) according to manufacturer’s instructions. Nasal or ocular swabs taken at 4 dpi, 6 dpi, 8 dpi, 10 dpi, and 12 dpi for each animal were vortexed (1 minute) and centrifuged (1,000 × *g*, 10 minutes, room temperature), and 100 µl of each supernatant was pooled for extraction.

### 2.9 Quantitative real-time RT-PCR

BTV segment 10 RNA was quantified by qRT-PCR as previously described (44) using a SuperScript™ III Platinum™ One-Step qRT-PCR kit with ROX (Thermo Fisher Scientific) and Stratagene Mx3005P thermocycler (Agilent Technologies, Santa Clara, CA, USA). Cycling conditions were 55°C for 30 minutes, 95°C for 10 minutes, and 45 cycles of 30°C for 30 seconds and 60°C for 1 minute. A 10-fold serial dilution of BTV-1 segment 10 RNA transcript was included on each plate to quantify BTV genome copies per ml blood (55) using MxPro software (Agilent Technologies).

### 2.10 Cytokine ELISAs

Chromogenic sandwich ELISAs were used to detect interferon-gamma (IFN-γ) and IL-4 cytokines in BTV-infected sheep serum as described (56), using CC330 (2 µg/ml)/CC302 (2 ng/ml) and CC314 (6 µg/ml)/CC313 (6 µg/ml) capture and biotinylated detection antibody pairs (Bio-Rad, Watford, UK), respectively. Ruminant sera (1:4, diluted in blocking buffer) were added in duplicate to plates alongside serially diluted recombinant purified IFN-γ protein (range 0.04 to 30 ng/ml; Bio-Rad) or *in vitro* expressed IL-4 protein (range 0.0005 to 5 ng/ml; The Pirbright Institute) (standards). Absorbances (450 nm and 690 nm) were measured using a Multiskan™ FC Microplate Photometer with SkanIt™ software version 4.1 (Thermo Fisher Scientific). Standard curves were generated using GraphPad Prism software (version 9.3.1), and four-parameter logistic regression was used to quantify IFN-γ and IL-4 from logarithm transformed optical densities (ODs) (450 nm minus non-specific 690 nm absorbance).

### 2.11 Detection of anti-BTV antibodies by ELISA

Sheep sera were screened for anti-BTV VP7 antibodies using the ID Screen^®^ BT cELISA (Innovative Diagnostics), according to the manufacturer’s instructions. A 50% reduction in percentage competition (S/N%) from sheep-matched pre-infection (−3 dpi to 0 dpi) serum was considered positive.

The ID Screen^®^ BT milk ELISA platform (Innovative Diagnostics) was modified to detect isotype-specific (IgM and IgG) anti-VP7 antibodies. All incubations were carried out at room temperature for 45 minutes. Sheep sera, diluted 1:100 in dilution buffer (IgM: dilution buffer 2 from ID Screen^®^ BT cELISA, IgG: 0.5% (w/v) sodium casein/0.05% (v/v) DPBS-T), were added in duplicate (100 µl/well) to plates for incubation. Plates were washed (3 × 300 µl/well, 0.05% (v/v) Tween 20 in DPBS), and 100 µl/well horseradish peroxidase (HRP)-conjugated (Lightning-Link^®^; Abcam, Cambridge, UK) rabbit anti-ovine IgM antibody (Bio-Rad, 1:2,000) or rabbit anti-bovine IgG Fc-HRP antibody conjugate (Sigma, 1:500) was added. Following incubation and washing, plates were developed using TMB substrate (Thermo Fisher Scientific) and stopped using 1 M sulphuric acid. Absorbances (450 nm and 620 nm) were measured as above, and ODs at 450 nm (minus non-specific 620 nm absorbance) were used to calculate a sample/negative (S/N) ratio by dividing average sample OD by average pre-infection (−3 dpi to 0 dpi) OD. OD ratios of 2.5 times the pre-infection OD or higher were considered positive. Selected matched pre- and post-infection sera (0 dpi and 12/18 dpi, respectively) from sheep with a range of low, mid, and high OD ratios (8.96–51.31 for IgM and 3.8–28.3 for IgG) were serially diluted twofold (range 1:100 to 1:204,800 post-infection sera; 1:100 to 1:800 pre-infection sera) and titrated on the IgM and IgG VP7 ELISAs as above. Antibody titres were expressed as the reciprocal highest dilution at which post-infection serum ODs exceeded matched pre-infection serum ODs at the 1:100 dilution.

ELISAs were developed to detect IgM- and IgG-specific anti-BTV NS2 antibodies similarly to above (except all incubations were 1 hour). For the IgG NS2 ELISA, 1 µg/ml in-house *Escherichia coli*-expressed recombinant BTV NS2 protein (rNS2; The Pirbright Institute) was coated overnight (4°C) onto Nunc™ Maxisorp™ clear 96-well plates (Thermo Fisher Scientific), and then the IgG VP7 ELISA protocol was followed. For the IgM NS2 ELISA, 4 µg/ml purified rabbit anti-ovine IgM antibody (Bio-Rad) was used to coat Maxisorp™ plates overnight (4°C). This was followed by sequential washing and incubation with sheep sera (1:100), rNS2 (4 µg/ml), and HRP-conjugated (Lightning-Link^®^) mouse anti-BTV NS2 antibody (1:200; Eurofins Technologies Ingenasa, Madrid, Spain), each diluted in dilution buffer 2. Plates were developed, stopped, and analysed as above.

### 2.12 Serum neutralisation test

A modified SNT protocol (57) was used to determine neutralising anti-VP2 antibody titres in ruminant serum using immunofluorescence-based detection of BTV infection. Ruminant sera (diluted 1:10 in diluent: DMEM-Glut with 1% P/S) was heat-inactivated (56°C, 30 minutes), added in quadruplicate to Nunc™ Microwell™ 96-well flat-bottom plates (Thermo Fisher Scientific), and serially titrated twofold from 1:10 to 1:5,120 (further if required). BTV-4 MOR2009/07 [KC2, BSR4] was diluted to 1,000 TCID_50_/ml (determined by titration) in a diluent, and 100 µl/well was added to titrated sera for incubation (37°C, 5% CO_2_, 1 hour). Vero cells were diluted (2 × 10^5^ cells/ml) in diluent containing 25% (v/v) heat-inactivated foetal bovine serum (FBS), and 50 µl/well was added for incubation (3 days, 37°C, 5% CO_2_). Quadruplicate positive (100 µl diluent/100 µl virus/50 µl cells) and negative (200 µl diluent/50 µl cells) controls were included on each plate. A viral titration was performed alongside each SNT batch to monitor viral titre.

Cells were fixed with 4% (v/v) paraformaldehyde/PBS (Thermo Fisher Scientific) for 30 minutes, washed in DPBS (3 × 150 µl/well), and then permeabilised for 20 minutes with 0.2% (v/v) Triton X-100 (Sigma) in PBS. Cells were sequentially washed and incubated with guinea pig anti-BTV hyperimmune serum (ORAB279, The Pirbright Institute) and then goat anti-guinea pig IgG (H&L)-AlexaFluor™ 488 secondary antibody (Thermo Fisher Scientific), diluted 1:2,000 and 1:250, respectively, in 0.5% (w/v) bovine serum albumin (Sigma) in DPBS (both incubations were at room temperature for 1 hour). The percentage of BTV-positive cells within each well was recorded using an Olympus CKX41 fluorescent microscope with a CoolLED pE300 microscope illuminator (Fullerscope, Banbury, UK). Neutralising anti-BTV VP2 antibody titres were calculated using a modified Reed–Muench calculation (58), as the reciprocal of the highest dilution in which viral replication was prevented in 50% of quadruplicate wells. Test plates were considered valid if a minimum of 50% of cells were observed to be infected with BTV across positive control wells (average). Anti-VP2 antibodies with “fully neutralising activity” were assessed through the inclusion of wells only where there was full protection (no BTV infection) of the cell monolayer. Anti-VP2 antibodies with only “partially neutralising activity” were assessed through additional inclusion of wells that were not fully neutralised, defined as wells in which <20% of cells in the monolayer were infected with BTV when compared to 50%–100% infection in the positive control.

### 2.13 Statistical analysis

Graphical plots and statistics were generated using GraphPad Prism software (version 9.3.1), except viral levels in *Culicoides* and *Culicoides* infection rate, which were analysed using lme4 package (59) in R (version 4.2.0) (60) and plotted using Matlab (The Mathworks Inc., version R2020b). Non-parametric Kruskal–Wallis tests were used to analyse differences between depletion and control groups in the following parameters at each time point (dpi): BTV genome copies per ml blood, serum IL-4/IFN-γ concentration, rectal temperature (°C) above pre-depletion/infection baseline, S/N% (cELISA), S/N OD ratios (IgM/IgG anti-VP7/NS2 serum antibodies), nAb titres, and the proportion of pre-depletion CD4^+^, CD8^+^, WC1^+^ γδ T cells, and CD21^+^ B cells in whole blood. Kruskal–Wallis tests were also used to investigate time-to-peak viraemia (dpi), time-to-peak (dpi) and highest IgM/IgG anti-VP7/NS2 antibody OD ratios, time to seroconversion, onset of viraemia (dpi at Cq 25), BTV genome copies per ml blood at peak viraemia, peak clinical scores, and time-to-peak clinical scores. Any statistically significant differences were further delineated using Dunn’s multiple comparisons tests to compare each T-cell depletion group (CD4^+^, CD8^+^, and WC1^+^ γδ) to mock, except whole-blood immune cell populations, which were compared to the transmission control. A non-parametric Mann–Whitney test was used to delineate statistical significance in BTV segment 10 genome copies per ml blood in sheep from each depletion group fed on high and low transmissibly infected (TI) *C. sonorensis.* A two-tailed non-parametric Spearman’s correlation identified correlations between log_10_ BTV genome copies per ml blood at the date of peak viraemia and clinical scores of sheep at 7 dpi and between S/N OD ratios and quantitative IgM/IgG VP7 antibody titres. A *p*-value of ≤0.05 was considered statistically significant for all tests.

A binomial family generalised linear mixed model (with logit link function) was used to compare the proportion of BTV-positive day 8 incubated *C. sonorensis* fed on sheep across depletion groups at peak viraemia. The response was whether the log_10_ BTV genome copies per ml blood for a *Culicoides* midge incubated for 8 days at 25°C, following imbibing of a potentially infectious blood meal, exceeded that recorded for day 0 incubated midges that fed on the same sheep. Explanatory variables were the depletion group (factor) and sheep (random effect, allowing for between-sheep variation). Infection rate of day 8 incubated *C. sonorensis* following feeding on BTV-infected sheep (CD4^+^, CD8^+^, or WC1^+^ γδ T cell or mock depleted) at peak viraemia was defined by a median viral load above that of matched D0 midges. A linear mixed model examined the relationship between viral load in the insect and that in the sheep on which the midge fed. The response was log_10_ BTV genome copies per ml in the midge. Explanatory variables were the depletion group (factor) and sheep (random effect, allowing for between-sheep variation). Analysis was repeated for midges tested immediately after feeding (D0) and after 8 days of incubation (D8).

### 2.14 Ethics statement

*In vivo* animal studies were carried out in accordance with the UK Animal Scientific Procedures Act (ASPA) 1986, which transposes the European Directive 2010/63/EU into UK National law. All studies were approved by the UK Home Office under Project License 70/7819 and P96CE012D. All procedures were reviewed and agreed upon locally by the Animal Welfare and Ethics Review Board at The Pirbright Institute.

## 3 Results

### 3.1 Circulating T-cell subsets were successfully depleted in sheep using specific mAbs to provide a unique insight into their function during *Culicoides-*borne BTV infection

Sheep were depleted of CD4^+^ (n = 7), CD8^+^ (n = 7), or WC1^+^ γδ (n = 7) T cells by intravenous administration of specific mAbs (GC1a, CC63, and CC15, respectively) over five consecutive days, initiated 2 days prior to infection with BTV (BTV-4 MOR2009/07) (**Figure 1**). A further seven sheep were “mock-depleted” prior to infection by administering an isotype-matched depletion control mAb (**Figure 1A**). Five control sheep were not depleted or infected with BTV but were co-housed with the infected sheep to monitor for potential contact transmission, to act as a negative control throughout the study, and to provide baseline immune cell dynamics (**Figure 1A**).

**Figure 2:**
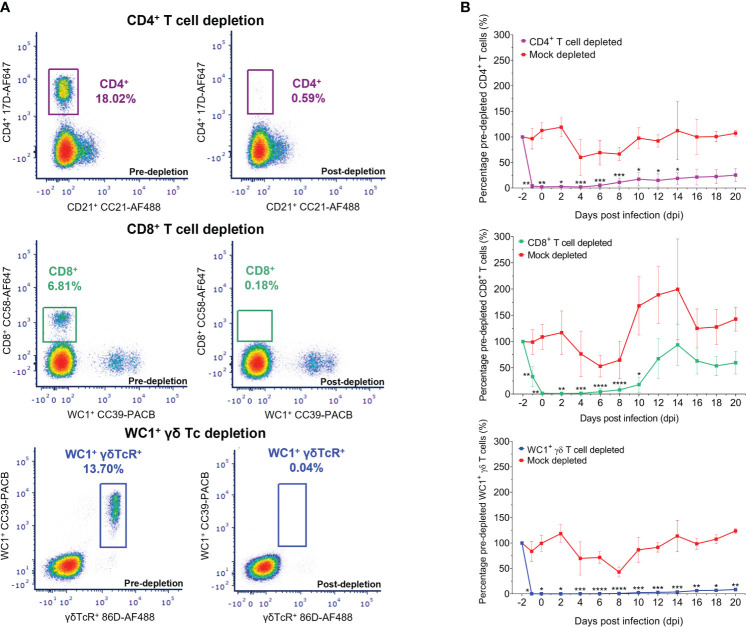
Depletion of circulating CD4^+^, CD8^+^, and WC1^+^/γδ TcR^+^ T cells in sheep using monoclonal antibodies as determined by flow cytometry. **(A)** Representative flow plots illustrating the percentage of CD4^+^ (purple), CD8^+^ (green), and WC1^+^/γδ TcR^+^ (blue) T cells of the total lymphocyte/monocyte population present before (pre-depletion) and after (post-depletion) intravenous injection of specific monoclonal antibodies (mAbs) as determined by flow cytometry. **(B)** Mean ( ± SD) percentage of baseline (pre-depletion) absolute CD4^+^, CD8^+^, or WC1^+^/γδ TcR^+^ T-cell numbers per ml blood detected in depleted and mock-depleted sheep during bluetongue virus (BTV) infection by flow cytometry. Percentage of baseline (pre-depletion) absolute cell numbers per ml blood at each day post infection (dpi) = post-depletion absolute cell number per ml blood/baseline absolute cell number per ml blood × 100. Mean values represent seven biological replicates for each treatment group, taken from mean triplicate technical replicates. Statistical significance in mean percentages of pre-depletion absolute cell numbers per ml blood for each T-cell depletion group compared to the transmission (negative) control group are given (≥0.0332 [*], ≥0.0021 [**], ≥0.0002 [***], ≤0.0001 [****]), as determined by a non-parametric Kruskal–Wallis test and *post-hoc* Dunn’s multiple comparisons.

In all sheep, the dynamics and/or depletion of each T-cell subset and the dynamics of B cells were monitored pre- and post-depletion and throughout BTV infection through detection of specific T-cell (CD4^+^, CD8^+^, WC1^+^, and ovine γδ T-cell receptor) and B-cell (CD21^+^) surface proteins in the live, singlet lymphocyte/monocyte population of whole blood using specific mAbs by flow cytometry (**Figure 2A**). On average, 238,472 events were collected for each blood sample, of which 96,700 were live singlet lymphocytes/monocytes, and, of these, 15,016 were CD4^+^ T cells, 11,867 were CD8^+^ T cells, 9,003 were WC1^+^/γδ TcR^+^ T cells, and 8,487 were CD21^+^ B cells. To account for the high variation in baseline (pre-depletion) absolute T- and B-cell numbers between individual sheep, post-depletion absolute cell numbers were normalised to matched baseline (pre-depletion) cell numbers for each sheep on each day. The absolute number of circulating CD4^+^, CD8^+^, and WC1^+^/γδ TcR^+^ T cells and CD21^+^ B cells were monitored before and after T-cell depletion and throughout BTV infection and are expressed here as a mean percentage of the baseline absolute cell numbers per ml blood for all animals within each treatment group (**Figure 2B**). The dynamics of T cells (CD4^+^, CD8^+^, and WC1^+^ γδ) in individual sheep of each treatment group during BTV infection are also given as absolute cell numbers per ml blood (**Supplementary Figure S2**) and a percentage of baseline absolute cell numbers per ml blood (**Supplementary Figure S3**).

Treatment with GC1a resulted in the depletion of circulating CD4^+^ T cells in all seven sheep, with an average of just 2.23% (range 0.13%–4.92%) baseline absolute CD4^+^ T-cell numbers remaining on the day of BTV infection (0 dpi) (**Figures 2B**; **Supplementary Figures S2**, **S3**). This represented, on average, 0.54% (range 0.05%–1.13%) of the lymphocyte/monocyte population, reduced from 21.28% (range 16.65%–27.39%) pre-depletion. CD4^+^ T-cell recovery was evident in the circulation of depleted sheep from 8 dpi to 10 dpi, on average to 25.35% (range 16.52%–39.84%) of the baseline (pre-depletion) absolute CD4^+^ T-cell numbers by 20 dpi. Circulating CD8^+^ T cells were severely depleted in all seven sheep treated with CC63, with an average of just 1.36% (range 0.00%–7.81%) baseline absolute CD8^+^ T-cell numbers remaining at 0 dpi (**Figure 2B**; **Supplementary Figures S2**, **S3**). This equated to an average of just 0.02% (range 0.00%–0.47%) of the lymphocyte/monocyte population, reduced from an average of 8.73% (range 5.13%–12.29%) before depletion. A substantial recovery and proliferative expansion of CD8^+^ T cells were observed in the blood of depleted sheep from 10 dpi to 14 dpi, with absolute CD8^+^ T-cell numbers reaching equivalent baseline values or higher in several sheep at 12 dpi and 14 dpi. Treatment with CC15 resulted in a substantial and prolonged depletion of circulating WC1^+^/γδ TcR^+^ T cells in all seven sheep throughout BTV infection, with on average just 0.09% (range 0.00%–0.026%) baseline WC1^+^/γδ TcR^+^ T cells remaining at 0 dpi (**Figure 2B**; **Supplementary Figures S2**, **S3**). This represented just 0.00%–0.02% of the lymphocyte/monocyte population, substantially reduced from 8.36% (range 4.48%–13.20%) pre-depletion. Notably, there were no changes in any T- or B-cell populations during the administration of the isotype-matched control mAb and no depletion of off-target immune cell populations resulting from the administration of GC1a, CC63, or CC15 mAbs.

*C. sonorensis* that had been exposed to a blood meal containing infectious quantities of BTV and then incubated were allowed to blood-feed on these sheep at the mid-point of ongoing T-cell depletion (0 dpi) to initiate and establish BTV infection in the continued absence of these specific T-cell subsets (**Figure 1**). All 28 sheep were successfully infected with BTV-4 MOR2009/07 using orally infected *C. sonorensis.* Initially, the impact of T-cell depletion was assessed on clinical disease observations during BTV infection.

### 3.2 T-cell depletion modulates clinical disease manifestation of BTV infection

**Figure 3:**
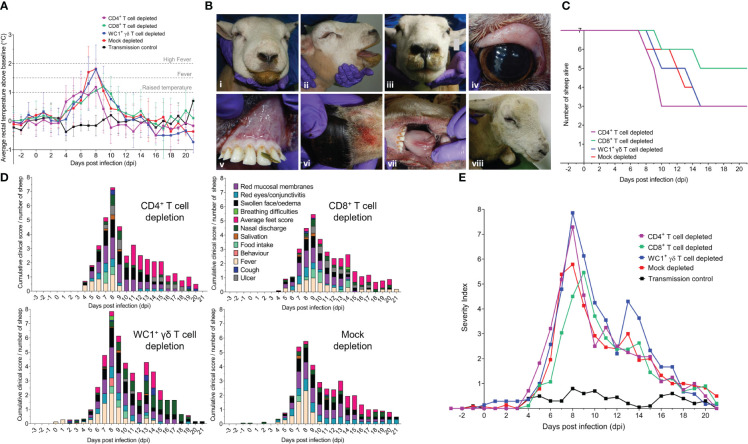
T-cell depletion modulates clinical disease outcomes of BTV-4 MOR2009/07 infection in sheep. **(A)** Mean ( ± SD) rectal temperatures (°C) above pre-depletion and pre-infection baseline temperatures in each of seven CD4^+^ (purple), CD8^+^ (green), WC1^+^ γδ (blue) T cell- and mock-depleted (red) sheep during infection with BTV-4 MOR2009/07. Raised temperature was defined as 1°C above animal-matched pre-infection baseline, fever by 1.5°C above baseline, and high fever by 2°C above baseline. Un-depleted and uninfected (negative control) sheep (black, n = 5) were included to monitor for contact transmission and general baseline temperature fluctuations. No statistical significance was identified in the means of each T-cell depletion group compared to the mock-depleted group at each time point (*p* > 0.05), as determined by a non-parametric Kruskal–Wallis test and *post-hoc* Dunn’s multiple comparisons. **(B)** Representative gross pathology of experimental bluetongue virus (BTV) infection in T cell- and mock-depleted sheep, characterised by **(i, ii)** severe facial oedema, **(iii)** nasal discharge, **(iv)** reddening of the eyes, **(v)** mucosal membranes, **(vi)** coronary band and **(vii)** tongue, and **(viii)** lameness and depression. **(C)** Survival curve of CD4^+^ (purple), CD8^+^ (green), WC1^+^ γδ (blue), and mock (red) T cell-depleted sheep over the duration of BTV-4 MOR2009/07 infection. **(D)** Mean cumulative daily clinical scores, as a proportion of the number of sheep alive, in CD4^+^, CD8^+^, or WC1^+^ γδ T cell- or mock-depleted sheep during infection with BTV-4 MOR2009/07. **(E)** Severity index of clinical disease in T cell/mock-depleted and control sheep during infection with BTV-4 MOR2009/07. Total daily clinical scores for all sheep within the CD4^+^ (purple), CD8^+^ (green), WC1^+^ γδ (blue), or mock (red) T-cell depletion groups were divided by the number of sheep alive per day. Negative (transmission) control sheep (n = 5) were uninfected and did not show gross clinical symptoms of BTV throughout the infection timeline.

All 28 BTV-infected sheep developed typical clinical signs of BT during infection. Importantly, we identified here that all three T-cell subsets (CD4^+^, CD8^+^, and WC1^+^ γδ) modulated clinical disease manifestation in BTV-infected sheep. When assessing the development of fever in BTV-infected sheep, we identified no statistically significant difference (*p* > 0.05) in rectal temperatures (above baseline values) at any time point during BTV infection between the T cell- and mock-depleted sheep (**Figure 3A**; **Table S1**). The inoculation of specific mAbs (−2 dpi to 2 dpi) did not induce fever in any of the animals. We observed an elevated temperature during this period only in sheep 27 (WC1^+^ γδ T cell depleted).

Sheep across all four BTV-infected treatment groups developed substantial facial oedema, nasal discharge, salivation, reddening of the eyes (conjunctivitis), mucosal membranes, coronary band and tongue, and lameness (**Figure 3B**). Four of the mock, CD4^+^, and WC1^+^ γδ T cell-depleted sheep and two CD8^+^ T cell-depleted sheep were euthanised prematurely (before 21 dpi), as they reached clinical humane endpoints of the study (**Figure 3C**). CD8^+^ T cell-depleted sheep were collectively alive longer (156 days, 89.1%) than sheep of the other groups. CD4^+^ T cell-depleted sheep collectively survived fewer days (124 days, 70.9%) than mock- and WC1^+^ γδ T cell-depleted sheep (both 135 days, 77.1%) despite an identical number being euthanised prematurely, indicative of a more acute clinical disease (**Figure 3C**).

The time-to-peak clinical disease was statistically significantly different between treatment groups (*p* = 0.010), with CD8^+^ T cell-depleted sheep reaching peak clinical scores later (*p* = 0.025) at 9 dpi (range 8–20 dpi) than the mock-depleted sheep at 8 dpi (range 7–9 dpi) (**Figure 3D**; **Supplementary Table S2**). However, there were no statistically significant differences in time-to-peak clinical scores between mock- and CD4^+^ or WC1^+^ γδ T cell-depleted sheep (8 dpi), *p* > 0.999 and *p* = 0.656, respectively (**Figure 3D**; **Supplementary Table S2**). There were also no statistically significant differences in the median non-cumulative clinical disease scores across each depletion group (*p* = 0.785; **Table S2**). Clinically, mouth ulceration was more prominent in CD4^+^ and CD8^+^ T cell-depleted sheep, haemorrhaging and/or oedema of the tongue in mock- and CD4^+^ T cell-depleted sheep, and depression across CD4^+^, CD8^+^, and WC1^+^ γδ T cell-depleted sheep. Nasal discharge was a particularly prominent feature of late infection in WC1^+^ γδ T cell-depleted sheep, whilst clinical scores of the feet (heat and redness) were the highest in the CD4^+^ T cell-depleted sheep (**Figure 3D**).

To account for the clinical severity and loss of clinical scores associated with sheep reaching their humane endpoint (which was particularly impactful at peak viraemia, i.e., peak clinical disease), we calculated a daily severity index to incorporate survival (days alive) into the clinical scoring. The daily severity index was calculated as the daily total clinical score for all animals within a group/daily number of sheep alive. Whilst the dynamics were similar across groups, the severity index peaked later (at 9 dpi) in the CD8^+^ T cell-depleted sheep compared to that of the mock, CD4^+^, and WC1^+^ γδ T cell-depleted sheep at 8 dpi (**Figure 3E**). CD4^+^ and WC1^+^ γδ T cell-depleted sheep also reached much higher severity indices (7.29 and 7.86, respectively) compared to the mock- and CD8^+^ T cell-depleted sheep (5.79 and 5.46, respectively; **Figure 3E**). Importantly, our data suggest that CD4^+^ T-cell depletion resulted in an acute onset of clinical disease, whilst WC1^+^ γδ T-cell depletion resulted in a more protracted presentation, which was likely also driven by the delayed clinical disease of sheep 9 (**Figure 3E**). When calculating a severity index score for all animals within each treatment group, defined as the cumulative daily severity index/total days alive, the index was the highest in the WC1^+^ γδ T cell-depleted sheep (1.981) followed by the CD4^+^ T cell- (1.619), mock- (1.539), and then CD8^+^ (1.417) T cell-depleted sheep. The negative control (uninfected) sheep (n = 5) remained clinically healthy throughout the experiment, except for mild non-descript conjunctivitis and nasal discharge observed in a few sheep at several time points (**Figure 3E**).

### 3.3 T cells do not influence BTV infection and replication dynamics

**Figure 4:**
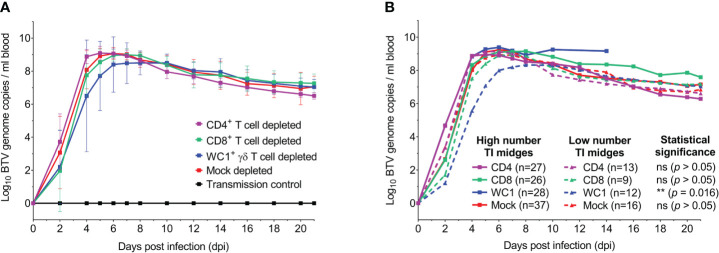
T cells do not influence bluetongue virus (BTV) infection dynamics in sheep. **(A)** Mean ( ± SD) log_10_ BTV genome copies detected per ml blood each in seven mock (red), CD4^+^ (purple), CD8^+^ (green), or WC1^+^ γδ (blue) T cell-depleted sheep during infection with BTV-4 MOR2009/07. BTV RNA was not detected in the blood of any uninfected (negative control) animals (black, n = 5), confirming contact transmission had not occurred. The means of each depletion group were not statistically significant (*p* > 0.05), as determined by a non-parametric Kruskal–Wallis test and *post-hoc* Dunn’s multiple comparisons. **(B)** Comparison of detectable BTV RNA (mean log_10_ BTV genome copies per ml blood) in mock (red), CD4^+^ (purple), CD8^+^ (green), and WC1^+^ γδ (blue) T cell-depleted sheep with either a high (n ≥ 18, solid lines) or low (n < 18) number of transmissibly infected (TI) midges (Cq < 25) blood-feeding to establish infection at 0 dpi. The average numbers of TI midges fed on sheep of each treatment group within each of the low and high TI midge categories are given in parentheses. WC1^+^ γδ T cell-depleted sheep had significantly earlier and higher detectable BTV RNA levels in the blood upon feeding by a higher (versus lower) number of TI midges (*p* = 0.016), suggesting a dose-dependent effect on viraemia linked to the WC1^+^ γδ T-cell subset. However, no significant differences were observed in mock, CD4^+^, or CD8^+^ T-cell depletion groups (*p* > 0.05). Statistical significance determined by non-parametric Mann–Whitney test.

To determine whether these observed clinical differences in T cell-depleted sheep were synonymous with differences in viral replication dynamics and/or viral load, the dynamics of BTV RNA in the blood during infection was next investigated. BTV RNA was first detected in sheep EDTA blood from 2 dpi to 5 dpi, with some biological variation observed between individuals within each BTV-infected treatment group (**Figure 4A**; **Supplementary Figure S4**). The onset of substantial viraemia (Cq < 25) was found to occur, on average, at 4 dpi for all treatment groups, except for one WC1^+^ γδ T cell-depleted sheep at 10 dpi (**Supplementary Figure S4**). No statistically significant differences were identified between treatment groups for either timing (*p* = 0.464) or the quantity of BTV RNA (defined as log_10_ BTV genome copies per ml blood) (*p* = 0.075). Time-to-peak viraemia was found to differ significantly in sheep between the treatment groups (*p* = 0.022), but only between CD4^+^ and CD8^+^ T cell-depleted sheep (*p* = 0.036). On average, peak viraemia occurred at 5 dpi for CD4^+^ T cell-depleted sheep, 6 dpi for mock-depleted sheep, and 7 dpi for CD8^+^ and WC1^+^ γδ T cell-depleted sheep (range from 5 dpi to 10 dpi); however, these differences were not statistically significant (*p* > 0.05). Average BTV RNA quantities at peak viraemia were also not statistically significantly different between treatment groups (*p* = 0.789). A moderate positive correlation was identified between average BTV RNA quantities at peak viraemia (*p* = 0.002, r = 0.611) and clinical scores at 7 dpi. Following peak viraemia, BTV RNA in the blood decreased to a steady plateau by 21 dpi across all treatment groups (**Figure 4A**); however, substantial BTV RNA remained (averaging 7.34 log_10_ BTV genome copies per ml blood). There were no significant differences in detectable BTV RNA levels in the blood at 21 dpi between the T cell- and mock-depleted sheep (*p* = 0.337). BTV RNA was not detected in the blood of any uninfected (negative control) sheep (**Figure 4A**) during the study, confirming that contact transmission had not occurred.

We next determined if the observed BTV viraemia dynamics were influenced by the number of TI *Culicoides* midges feeding on the sheep to establish infection. Here, we inferred TI midges as those with a BTV segment 10 qRT-PCR Cq value of less than 25 (42). Comparing the viraemia profile in sheep infected by a low (n < 18) and high (n ≥ 18) number of TI midges at 0 dpi (**Figure 4B**), we observed a significant difference in the viraemia profiles of WC1^+^ γδ T cell-depleted sheep (*p* = 0.016), but not mock, CD4^+^, or CD8^+^ T cell-depleted sheep (*p* = 0.744, 0.779, and 0.106, respectively). Our data demonstrate that only in the absence of WC1^+^ γδ T cells did a higher number of blood-feeding TI *Culicoides* midges lead to earlier and higher quantities of detectable BTV RNA in the blood. To confirm that this statistically significant difference was not due to the substantially delayed viraemia of sheep 9 (WC1^+^ γδ T cell depleted) alone (**Supplementary Figure S4**), we excluded sheep 9 from the dataset and re-ran the statistical analysis, confirming that for WC1^+^ γδ T cell-depleted sheep, the viraemia profile was still statistically significantly different between sheep infected by a low or high number of TI midges. A comparable number of TI midges (n = 17) were found to have blood-fed on sheep 9 when compared to other sheep within and across treatment groups, suggesting that this observed delay in viraemia was not driven by the numbers of infected *Culicoides* feeding.

Nasal and ocular cavities of all BTV-infected sheep were swabbed during peak viraemia for the presence of BTV RNA as an indicator of nasal or ocular viral shedding; however, only low-level BTV RNA was detectable by qRT-PCR (mean Cq 35.11 and 38.13, respectively; **Supplementary Table S3**) and likely resulted from local viral replication or the presence of detectable BTV in residual blood and/or cells in nasal/ocular cavities upon swabbing. BTV RNA was not detectable in swabs taken from the five co-housed uninfected sheep, again confirming the absence of contact transmission.

### 3.4 T cells do not influence BTV infection rate of *C. sonorensis* feeding on peak viraemic sheep

**Figure 5:**
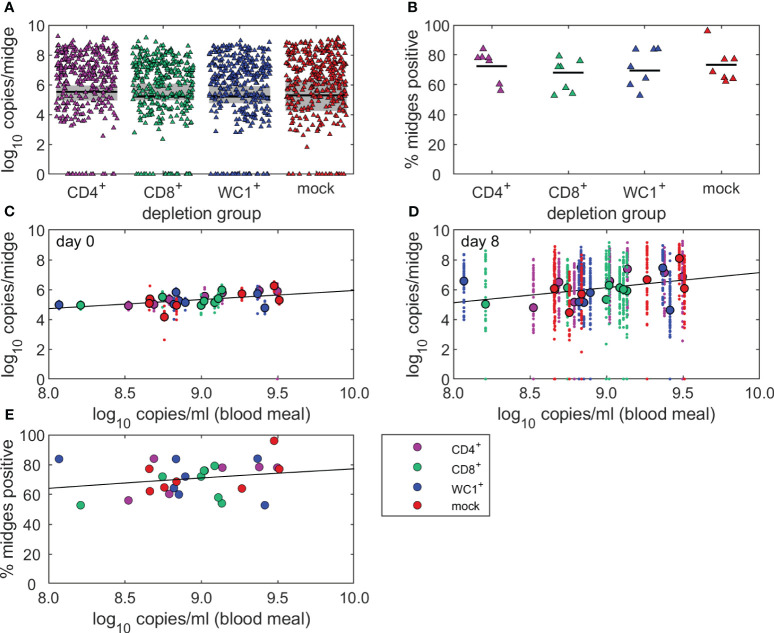
T cells do not influence bluetongue virus (BTV) infection rate of *Culicoides sonorensis* feeding on viraemic sheep. **(A, B)** Uninfected, colonised *C*. *sonorensis* were fed on BTV-infected sheep (n = 28) at peak viraemia to determine whether absence of individual T-cell subsets (CD4^+^, CD8^+^, and WC1^+^ γδ) influenced onwards transmission of BTV to a susceptible *Culicoides* vector. **(A)** Log_10_ BTV genome copies in individual engorged female *C*. *sonorensis* midges incubated for 8 days (triangles) at 25°C following blood-feeding on CD4^+^ (purple, n = 345), CD8^+^ (green, n = 345), or WC1^+^ γδ (blue, n = 338) T cell- or mock-depleted (red, n = 357) sheep at peak viraemia during infection with BTV-4 MOR2009/07. Baseline viral uptake (median, black line; range, grey shading) was determined by quantifying the log_10_ BTV genome copies per midge in identically fed *C*. *sonorensis* (n = 88, 80, 88, and 104, respectively) processed immediately (day 0) following feeding on the sheep. **(B)** Variation in infection rate (proportion of BTV-positive midges, %) of *C*. *sonorensis* fed on individual BTV-4 infected sheep (dots; median, black line) following CD4^+^ (n = 7), CD8^+^ (n = 7), or WC1^+^ γδ (n = 7) T cell or mock (n = 7) depletion. No statistically significant differences were identified in infection rate between depletion groups using median viral uptake of matched day 0 midges as cut-off for viral replication, as determined using binomial family generalised linear mixed model with logit link function. **(C, D)** Linear regression of the relationship between viral load in CD4^+^, CD8^+^, or WC1^+^ γδ T cell- and mock-depleted sheep on the day of midge feeding (peak viraemia) and viral load in the **(C)** day 0 and **(D)** day 8 incubated *C*. *sonorensis*. Log_10_ BTV genome copies detected in individual insects (dots) and the mean of all insects fed on individual sheep (circles) are shown alongside the best-fit linear regression line. **(E)** Logistic regression of the relationship between infection rate (proportion of BTV-positive midges, %) in *C. sonorensis* fed on individual CD4^+^ (n = 7), CD8^+^ (n = 7), or WC1^+^ γδ (n = 7) T cell- or mock-depleted (n = 7) sheep (circles) and the viral load (log_10_ BTV genome copies per ml blood) in individual sheep at the time of *Culicoides* feeding. The fitted black line demonstrates a positive, but not significant (*p* = 0.200), relationship between *Culicoides* infection rate and infectious blood meal.

To determine whether the absence of individual T-cell subsets (CD4^+^, CD8^+^, or WC1^+^ γδ) influenced onwards transmission of BTV to a susceptible *Culicoides* vector, we fed uninfected, colonised *C. sonorensis* on each BTV-infected sheep (n = 28) at peak viraemia. Midges were fed on sheep at either 6 dpi or 7 dpi, except for sheep 9, which was exposed to *C. sonorensis* feeding at 10 dpi due to its substantially delayed peak viraemia. There was little variation in the average viral load of sheep across each treatment group at the time of *C. sonorensis* blood-feeding, with 9.01 (CD4), 8.90 (CD8), 8.89 (WC1), and 9.02 (mock) log_10_ BTV genome copies per ml blood. Following blood-feeding on CD4^+^, CD8^+^, and WC1^+^ γδ T cell- or mock-depleted sheep, a total of 360 engorged female *C. sonorensis* individuals (n = 88, 80, 88, and 104, respectively) were immediately processed (day 0) by qRT-PCR to quantify the baseline of how much virus (inferred by the viral genome) each blood-feeding *Culicoides* had taken up (**Figure 5A**). A further 1,385 engorged female *C. sonorensis* (n = 345, 345, 338, and 357, respectively, of CD4^+^, CD8^+^, and WC1^+^ γδ T cell- and mock-depleted sheep) were incubated for 8 days at 25°C to complete the BTV extrinsic incubation period and were then processed to assess viral load and determine whether viral replication had occurred (**Figure 5A**). As some variability was observed in BTV uptake by *C. sonorensis* between individual sheep (range 5.4–5.7 log_10_ BTV genome copies per midge), an individual sheep-specific cut-off was used to accurately define BTV replication in day 8 incubated midges. In this case, the median viral uptake in sheep-matched day 0 midges (log_10_ BTV genome copies per midge) was used (**Figure 5A**).

There was no significant difference (*p* = 0.695) in the number of *C. sonorensis* developing a transmissible infection (n = 85, 95, and 92 versus n = 111, respectively) after feeding on CD4^+^, CD8^+^, or WC1^+^ γδ T cell- compared to mock-depleted sheep (**Figure 5A**). There was high variation in both initial viral uptake and the proportion (%) of *C. sonorensis* becoming infected (infection rate) after feeding on individual sheep within each depletion group (**Figure 5B**). Whilst this was not linked to the T-cell depletion itself (*p* = 0.758), the viral uptake (log_10_ BTV genome copies per midge) in *C. sonorensis* fed on peak viraemic sheep did significantly increase in line with viral load in the sheep blood meal (day 0, *p* = 0.005; day 8, *p* = 0.030) (**Figures 5C, D**). Whilst the infection rate did also increase with viral load in the sheep blood meal (**Figure 5E**), this was not statistically significant (*p* = 0.200).

### 3.5 CD4^+^ and WC1^+^ γδ T-cell depletion results in a lack of CD8^+^ T-cell expansion after peak viraemia

**Figure 6:**
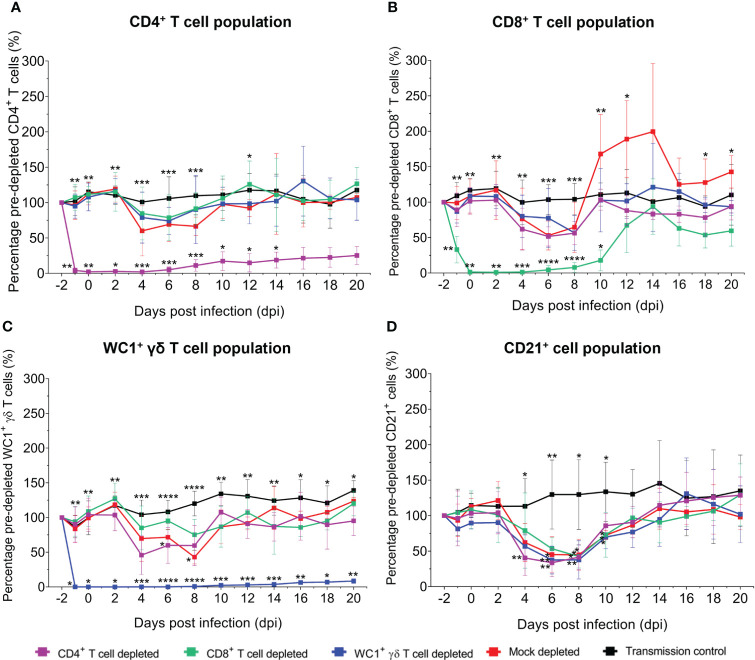
Monitoring the depletion of CD4^+^, CD8^+^, and WC1^+^ γδ T-cell subsets and dynamics of immune cell populations during bluetongue virus (BTV) infection in sheep by flow cytometry. Mean ( ± SD) percentage of baseline (pre-depletion) absolute **(A)** CD4^+^, **(B)** CD8^+^, **(C)** WC1^+^/γδ TcR^+^ T cell, or **(D)** CD21^+^ B-cell numbers per ml blood detected in sheep during BTV infection by flow cytometry following depletion of CD4^+^ (purple), CD8^+^ (green), or WC1^+^ γδ (blue) T cells or mock depletion (red), with specific monoclonal antibodies (mAbs). Percentage of baseline (pre-depletion) absolute cell numbers per ml blood at each day post infection (dpi) = post-depletion absolute cell number per ml blood/baseline absolute cell number per ml blood × 100. Mean values represent seven biological replicates for each treatment group, taken from mean triplicate technical replicates. Five un-depleted and uninfected (negative control) sheep (black) were also included to serve as a baseline for immune cell dynamics. Statistical significance in mean percentages of pre-depletion absolute cell numbers per ml blood for each depletion group compared to the transmission control is given (≥0.0332 [*], ≥0.0021 [**], ≥0.0002 [***], ≤0.0001 [****]), as determined by a non-parametric Kruskal–Wallis test and *post-hoc* Dunn’s multiple comparisons.

To investigate whether depletion of specific T-cell subsets influenced the dynamics of other non-depleted immune cell populations (CD4^+^, CD8^+^, and WC1^+^/γδ TcR^+^ T cells and CD21^+^ B cells) during BTV infection in sheep, we performed immunophenotyping of ovine whole blood at regular intervals during infection and assessed immune cell populations by flow cytometry (**Figures 6A–D**). A transient pan-lymphopenia was observed in BTV-infected sheep of all treatment groups (**Figures 6A–D**; **Supplementary Figures S2**, **S3**). The timing of this lymphopenia was closely associated with quantities of detectable BTV RNA in the blood, beginning on average at 4 dpi at the onset of viraemia and peaking at the height of viraemia, with lymphocyte populations generally recovering upon the plateau of the blood viral load. The CD21^+^ B-cell population demonstrated the most substantial and significant losses, observed transiently, but consistently, across BTV-infected sheep of all treatment groups from 4 dpi to 10 dpi (**Figure 6D**). Mock-depleted sheep had significantly lower CD21^+^ B cells at 6 dpi, 8 dpi, and 10 dpi compared to uninfected (negative control) sheep (*p* = 0.020, 0.029, and 0.018). This transient reduction in CD21^+^ B cells was significantly earlier from 4 dpi to 8 dpi (*p* = 0.002, 0.003, and 0.034) in CD4^+^ T cell-depleted sheep compared to uninfected (negative) control sheep (**Figure 6D**).

CD4^+^, CD8^+^, and WC1^+^ γδ T cells decreased from baseline (pre-depletion) numbers at 4 dpi, 6 dpi, and 8 dpi in BTV-infected sheep of each treatment group (**Figures 6A–C**). Whilst WC1^+^ γδ T cells were significantly lower compared to the uninfected (negative control) sheep at 6 dpi for the CD4^+^ T cell-depleted sheep (*p* = 0.049) and 8 dpi for the mock-depleted sheep (*p* = 0.018), the biological relevance of these single data point differences is uncertain given the inherent variation between sheep during the infection time course. A transient expansion in the CD8^+^ T-cell population was observed later from 10 dpi to 14 dpi in the mock-depleted sheep but was absent in the CD4^+^ or WC1^+^ γδ T cell-depleted sheep (**Figure 6B**). Whilst CD4^+^ T-cell depletion led to an earlier loss of the WC1^+^ γδ T-cell population compared to the mock-depleted sheep, CD8^+^ T-cell depletion resulted in no loss of this population during peak infection. The administration of specific mAbs to deplete individual T-cell subsets did not impact the non-depleted immune cell populations (CD4^+^, CD8^+^, or WC1^+^ γδ T cells or CD21^+^ cells), with the observed lymphopenia occurring after this depletion window of −2 dpi to 2 dpi.

### 3.6 Abrogation of specific T-cell subsets induces interferon-gamma and interleukin-4 otherwise absent during BTV infection

**Figure 7:**
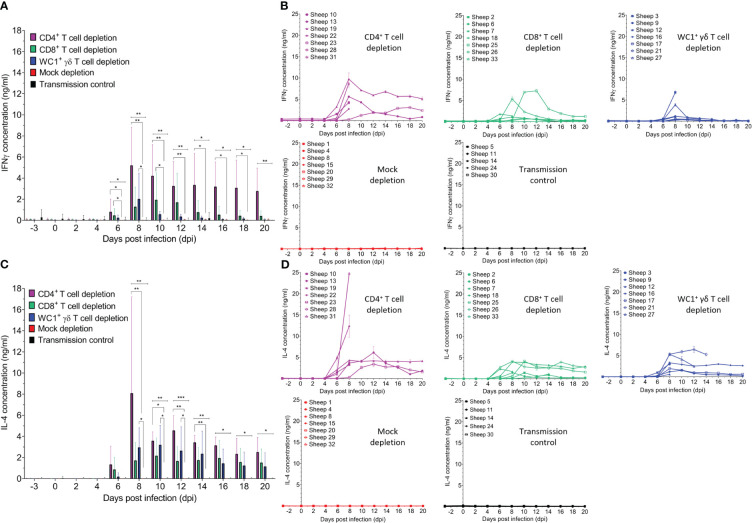
Specific T-cell subset depletion induces IFN-γ and IL-4 responses otherwise absent during bluetongue virus (BTV) infection. Mean ( ± SD) **(A)** IFN-γ and **(C)** IL-4 concentrations (ng/ml) in the serum of CD4^+^ (purple, n = 7), CD8^+^ (green, n = 7), or WC1^+^ γδ (blue, n = 7) T cell- or mock-depleted (red, n = 7) sheep during infection with BTV-4 MOR2009/07 as determined by quantitative sandwich ELISA. Mean IFN-γ and IL-4 concentrations in the serum of un-depleted and uninfected (negative control) sheep (n = 5) are also shown. Statistical significance (*p* < 0.05) in means of each T-cell depletion group compared to that of the mock-depleted control is given (≥0.0332 [*], ≥0.0021 [**], ≥0.0002 [***], ≤ 0.0001 [****]), as determined by a non-parametric Kruskal–Wallis test and *post-hoc* Dunn’s multiple comparisons. **(B)** IFN-γ and **(D)** IL-4 concentrations (ng/ml) detected in the serum of individual sheep of the CD4^+^, CD8^+^, WC1^+^ γδ T cell- and mock-depleted groups as well as the transmission control group.

Previous *in vivo* and *in vitro* BTV studies have implicated both pro-inflammatory/anti-viral T helper type 1 (Th1) and anti-inflammatory type 2 (Th2) cytokines; however, responses observed were highly variable and dependent on the infecting BTV strain, virulence, and/or individual host variation. As cytokines are known to facilitate T-cell effector functions, we next examined if the depletion of specific T-cell subsets impacted serum dynamics of representative Th1, IFN-γ, and Th2, interleukin-4 (IL-4), and cytokine during BTV infection (**Figure 7**). Whilst mock-depleted sheep had negligible detectable serum IFN-γ (**Figure 7B**) and IL-4 (**Figure 7D**) throughout BTV infection (mean: 0.028 ng/ml and 0.006 ng/ml, respectively), both cytokines were detected in all T cell-depleted sheep typically from 6 dpi to 8 dpi (around peak viraemia), except sheep 28 (CD4^+^ T cell depleted), which was euthanised at 7 dpi.

Serum IFN-γ was significantly higher in CD4^+^ (6–18 dpi), CD8^+^ (6–10 dpi), and WC1^+^ γδ (6–8 dpi) T cell-depleted sheep compared to mock-depleted sheep (**Figure 7A**). CD4^+^ T cell-depleted sheep had a higher average peak in serum IFN-γ concentration (5.684 ng/ml) compared to the CD8^+^ and WC1^+^ γδ T cell-depleted sheep (2.444 and 2.047 ng/ml, respectively). Serum IFN-γ in nearly all sheep declined to pre-infection levels except for two CD4^+^ (#19/23) and one CD8^+^ (#25) T cell-depleted sheep, which had detectable IFN-γ levels of 5.122 ng/ml, 2.366 ng/ml, and 1.253 ng/ml, respectively, at 20 dpi. CD4^+^ and WC1^+^ γδ (but not CD8^+^) T cell-depleted sheep also had significantly higher serum IL-4 than the mock-depleted sheep from 8 dpi to 14 dpi (*p* = 0.002 and 0.009) and 8 dpi to 12 dpi (*p* = 0.011 and 0.049), respectively (**Figure 7C**). Whilst CD4^+^ T cell-depleted sheep had a much higher mean peak in serum IL-4 than CD8^+^ and WC1^+^ γδ T cell-depleted sheep (9.026 ng/ml, 3.161 ng/ml, and 2.639 ng/ml, respectively), this was due to high spikes in two CD4^+^ T cell-depleted sheep (#31/22) at peak infection (24.79 and 12.32 ng/ml) prior to euthanasia. Serum IL-4 tended to peak and then plateau to 20 dpi in most sheep. Notably, there were no observed changes in serum IFN-γ or IL-4 prior to infection in response to the T-cell depletion (or administration of mAbs) in any sheep, thereby highlighting that the increase in serum IFN-γ and IL-4 observed during infection correlated to BTV viraemia in T cell-depleted sheep.

No detectable changes in serum IFN-γ (mean 0.009 ng/ml) or IL-4 (mean 0.059 ng/ml) were identified in the uninfected (negative control) sheep during the experiment, except for an elevated IFN-γ response (mean 0.719 ng/ml, range 0.329–1.566 ng/ml), which was present prior to the study and throughout the time course (−3 dpi to 14 dpi) in sheep 30, likely caused by a pre-existing bacterial/viral infection.

### 3.7 BTV-infected sheep develop detectable anti-VP7 antibodies earlier in the absence of CD4^+^ T cells

**Figure 8:**
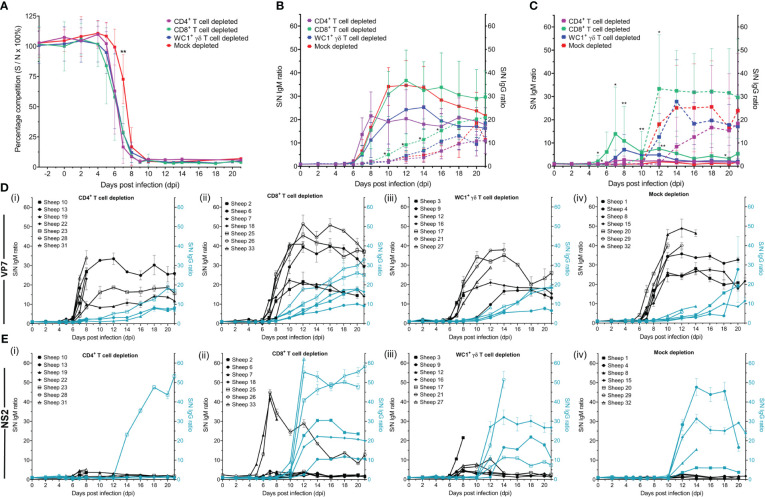
Host immunoglobulin subclasses against bluetongue virus (BTV) VP7 and NS2 proteins respond differently to T-cell depletion. **(A)** Percentage competition (S/N% ± SD) of anti-BTV VP7 antibodies in serum of CD4^+^ (purple, n = 7), CD8^+^ (green, n = 7), or WC1^+^ γδ (blue, n = 7) T cell- or mock-depleted (red, n = 7) sheep during infection with BTV-4 MOR2009/07 as determined by the ID Screen^®^ BT cELISA. Competition was calculated as a percentage of the test sample (S) optical density (OD) from the negative (N) control OD, with a 50% reduction in S/N% from that of the average sheep-matched pre-infection (−3 dpi to 0 dpi) serum considered positive for anti-VP7 antibodies. CD4^+^ T cell-depleted sheep seroconverted significantly earlier than mock-depleted sheep (*p* = 0.023 [**]), as determined by a non-parametric Kruskal–Wallis test and *post-hoc* Dunn’s multiple comparisons. **(B)** IgM (solid lines) and IgG (dotted lines) anti-VP7 or **(C)** anti-NS2 antibodies detected in CD4^+^ (purple), CD8^+^ (green), or WC1^+^ γδ (blue) T cell- or mock-depleted (red) sheep during infection with BTV-4 MOR2009/07. Absorbance at 450 nm is presented as a ratio of average sample (S) to negative (average pre-infection, −3 dpi to 0 dpi) (N) OD values performed in duplicate, with error bars representing standard deviation (SD). Statistical significance in S/N OD ratio at each time point is given (≥0.0332 [*], ≥0.0021 [**], ≥0.0002), as determined by a non-parametric Kruskal–Wallis test. For those found to be significant, a *post-hoc* Dunn’s multiple comparisons test was carried out to determine whether T-cell depletion groups significantly differed from the mock-depleted group. **(D)** S/N OD ratios ( ± SD) for IgM (black) and IgG (blue) anti-VP7 and **(E)** anti-NS2 antibodies detected in the serum of individual sheep of the **(i)** CD4^+^, **(ii)** CD8^+^, or **(iii)** WC1^+^ γδ T cell- or **(iv)** mock-depleted groups during BTV infection.

All BTV-infected sheep had a detectable anti-VP7 antibody response by commercially available diagnostic BT competition ELISA (cELISA), except sheep 32 (mock depleted) which was euthanised at 7 dpi (**Figure 8A**). Seroconversion of anti-VP7 antibodies occurred earlier in T cell-depleted sheep than mock-depleted sheep (**Figure 8A**), on average at 7 dpi (range 5–8 dpi) rather than 8 dpi (86%); however, this was only statistically significant for the CD4^+^ T cell-depleted sheep (*p* = 0.023).

### 3.8 CD8^+^ T-cell depletion results in earlier class switching of IgG VP7 antibodies

To determine whether the absence of specific T-cell subsets translated to a delay in class switching of anti-VP7 antibodies to an IgG isotype, we modified the diagnostic BT ELISA to enable the detection of VP7-specific IgM and IgG antibodies and used these isotype-specific BTV VP7 ELISAs to screen selected time-course sera from the T cell/mock-depleted sheep (**Figures 8B, D**). Due to the high variability in background OD values between individual sheep, we calculated a sample (S)/negative (N) antibody ratio for each antibody subclass at each dpi for each animal, as follows: post-infection serum OD value/average pre-infection (−3 dpi to 0 dpi) serum OD value × 100.

IgM anti-VP7 antibodies were detected typically just after peak viraemia, on average from 7 dpi (range 5–10 dpi), in all BTV-infected sheep, except in the case of sheep 12, which was euthanised at 9 dpi (**Figures 8B, D**). Time of seroconversion with IgM anti-VP7 antibodies was not significantly different between the T cell- and mock-depleted sheep (*p* = 0.067). IgM anti-VP7 antibodies typically peaked between 7 dpi and 18 dpi; however, there were no statistically significant differences in time-to-peak ratios between T cell- and mock-depleted sheep (*p* = 0.410) (**Figure 8D**). The mean peak S/N IgM anti-VP7 antibody ratios were lower in the CD4^+^ and WC1^+^ γδ T cell-depleted sheep compared to the mock- and CD8^+^ T cell-depleted sheep (**Figures 8B, D**). However, there was substantial variation in these ratios between individual sheep regardless of treatment group, and differences were not statistically significant (*p* = 0.276). Anti-VP7 IgG antibodies were detected after the specific IgM antibodies during BTV infection, typically from 11 dpi (range 8–16 dpi) and increasing up to 21 dpi (or the humane end points) in nearly all sheep (**Figures 8B, D**). There was a significant difference in the time to seroconversion of IgG anti-VP7 antibodies (*p* = 0.043), with CD8^+^ T cell-depleted sheep producing IgG significantly earlier (8 dpi or 10 dpi) than mock-depleted sheep (10 dpi to 14 dpi) (*p* = 0.044), but later reaching comparable S/N ratios to mock-depleted sheep (*p* = 0.144; **Figures 8B, D**).

To determine whether the S/N IgM and IgG anti-VP7 antibody ratios were representative of quantitative antibody titres, selected pre- and post-infection sera from sheep within each BTV-infected treatment group with low-, mid-, and high-range ratios were titrated on each subclass ELISA. S/N IgM (**Supplementary Figure S5A**) and IgG (**Supplementary Figure S5B**) anti-VP7 antibody ratios were each found to have a significant strong positive correlation to equivalent IgM or IgG anti-VP7 antibody titres (*p* = 0.001 and 0.001), with correlation coefficients of 0.717 and 0.788, respectively. We can therefore infer that S/N antibody ratios broadly correlated to antibody quantities.

All sheep, except those euthanised before the end of the study, generated a uniform anti-VP7 IgM response and subsequent class-switched IgG response upon infection with BTV. Immunoglobulin production to BTV VP7 was largely found to be independent of the depletion of specific T-cell subsets, with the exception that the absence of CD8^+^ T cells resulted in earlier class switching to IgG in infected sheep, which may indicate a role for this subset in BTV pathogenesis.

### 3.9 CD4^+^ T cells appear to be important for IgG anti-NS2 antibody production in BTV-infected sheep

Whilst host immune responses to VP7 form the basis of the current diagnostic BT cELISA, those against non-structural BTV proteins (e.g., NS2) generated upon viral replication within a cell are underexplored. Whilst BTV-infected ruminants have the capacity to generate antibodies to all BTV proteins, their presence and dynamics during infection have yet to be characterised. In this study, we developed novel ELISAs to detect anti-NS2 IgM and IgG antibody responses in ruminant serum using bacterially expressed recombinant NS2 protein. Here, for the first time, we present their dynamics during BTV infection and demonstrate that CD4^+^ T cells appear to be important for IgG NS2 antibody production in sheep.

Anti-NS2 IgM antibodies were initially detectable from between 5 dpi and 12 dpi (**Figures 8C, E**), but not in all BTV-infected sheep, unlike anti-VP7 antibodies. Of the seven CD4^+^ T cell-depleted sheep, three had detectable anti-NS2 IgM antibodies at 7 dpi (n = 2) and 8 dpi (n = 1), but notably with very low S/N ratios (4.15, 5.05, and 3.45, respectively); two of the sheep did not seroconvert, and two sheep were euthanised at 7 dpi and 9 dpi (**Figure 8E**i). Of the seven CD8^+^ T cell-depleted sheep, all seroconverted for anti-NS2 IgM antibodies on average at 8 dpi (range 5–12 dpi) with an average S/N OD ratio of 15 (range 3.35–44.9) (**Figure 8E**ii). Anti-NS2 IgM antibodies were also detectable in all WC1^+^ γδ T cell-depleted sheep, typically seroconverting at 8 dpi (range 7–8 dpi), except one sheep, which was euthanised at 9 dpi, with S/N OD ratios averaging 9 (range 4.15–21.5) (**Figure 8E**iii). Importantly, of the seven mock-depleted sheep, only one was found to seroconvert for anti-NS2 IgM antibodies (at 12 dpi, S/N OD ratio of 2.9), with four euthanised between 7 dpi and 14 dpi and two which had not seroconverted by 21 dpi (**Figure 8E**iv).

Of the sheep that had detectable IgM anti-NS2 antibodies, we found no statistically significant differences in time-to-seroconversion or time-to-peak antibody ratios when comparing T cell- and mock-depleted sheep (*p* = 0.591 and 0.448, respectively). We did find significant differences in the IgM anti-NS2 antibody S/N ratios of sheep at 5 dpi, 7 dpi, 8 dpi, 10 dpi, 12 dpi, and 20 dpi (**Figure 8C**), with CD8^+^ T cell-depleted sheep having significantly higher ratios to mock-depleted sheep at 7 and 10 dpi (*p* = 0.036 and 0.046) and WC1^+^ γδ T cell-depleted sheep at 7 dpi, 8 dpi, and 10 dpi (*p* = 0.048, 0.003, and 0.003). Notably, two CD8^+^ (#7/26) and one WC1^+^ γδ (#3) T cell-depleted sheep had greater IgM anti-NS2 antibody responses to all other sheep (**Figure 8E**ii–iii), likely contributing to these observed differences. There were no significant differences in IgM NS2 antibody ratios between mock- and CD4^+^ T cell-depleted sheep during BTV infection (*p* > 0.05). There was also no significant difference in the peak OD ratios themselves (*p* = 0.188). By 21 dpi, IgM anti-NS2 antibodies were no longer detectable in most of the BTV-infected sheep.

Strong anti-NS2 IgG antibody responses were observed in most, but not all, BTV-infected sheep typically from 12 dpi (range 10–14 dpi). Whilst high IgG anti-NS2 antibody responses were observed in six of seven CD8^+^ T cell-depleted sheep (the other euthanised at 8 dpi), five of seven WC1^+^ γδ T cell-depleted sheep (the remaining two sheep euthanised at 8 dpi), and five of seven mock-depleted sheep (two remaining sheep euthanised at 8 and 10 dpi) in the serum we tested, only one of the CD4^+^ T cell-depleted sheep we tested had detectable IgG anti-NS2 antibodies, despite three surviving to 21 dpi. Of the three surviving CD4^+^ T cell-depleted sheep, two did not produce any anti-NS2 IgG antibodies, whilst in the third sheep, the production of NS2-specific IgG was delayed to 14 dpi (**Figure 8E**i), compared to 11 dpi and 12 dpi (on average) in the responding CD8^+^ and mock/WC1^+^ γδ T cell-depleted sheep, respectively. Whilst we identified a significant difference in time to seroconversion across the BTV-infected sheep (*p* = 0.007), the low number of sheep within each treatment group at these later time points likely precluded identifying which groups differed (*p* > 0.05). S/N IgG anti-NS2 antibody ratios were significantly different between treatment groups at 5 dpi and 12 dpi (*p* = 0.024 and 0.020), with CD8^+^ T cell-depleted sheep having significantly higher antibody ratios than WC1^+^ γδ T cell-depleted sheep at 5 dpi (*p* = 0.014) and CD4^+^ T cell-depleted sheep at 12 dpi (*p* = 0.024). However, there were no significant differences between antibody ratios of T cell- and mock-depleted sheep (*p* > 0.05). Despite the observed delay in detectable IgG anti-NS2 antibodies in sheep 23 (CD4^+^ T cell depleted), comparable OD ratios (average 53.2) were reached to those of the other treatment groups by 21 dpi. IgG anti-NS2 antibodies typically plateaued or were in decline towards the end of the experiment (**Figures 8C, E**i–iv).

In this study, we observed that IgM anti-NS2 antibodies were less uniformly produced when compared to IgM anti-VP7 antibodies, with only a few animals (n = 2 and 3, respectively) in each of the CD8^+^ and WC1^+^ γδ T-cell depletion groups reaching substantial (>6) S/N IgM anti-NS2 antibody ratios. In contrast, IgG anti-NS2 antibody production was much more uniform in those sheep surviving beyond 12 dpi, and, here, only depletion of CD4^+^ T cells resulted in a delayed or absent detection of IgG anti-NS2 antibodies, suggesting that this subset is likely important for timely production of IgG NS2 antibodies during BTV infection in sheep.

### 3.10 CD4^+^ T-cell depletion appears to impair the neutralising antibody response to BTV infection in sheep

**Figure 9:**
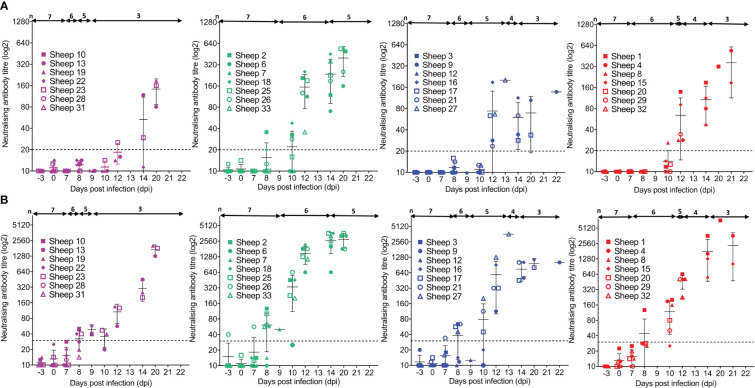
Neutralising anti-VP2 antibody titres in T cell- or mock-depleted sheep during bluetongue virus (BTV) infection. Neutralising antibody titres in CD4^+^ (purple, n = 7), CD8^+^ (green, n = 7), or WC1^+^ (blue, n = 7) T cell- and mock-depleted (red, n = 7) sheep during infection with BTV-4 MOR2009/07 as determined by immunofluorescence-based serum neutralisation test when assessing antibodies with **(A)** fully neutralising activity (absence of BTV infection/fully protected cells across the monolayer) and **(B)** fully and partially neutralising activity (with partially neutralising activity defined as wells with less than 20% BTV infection remaining in the cell monolayer compared to 50%–100% infection in the positive control). Dots represent anti-VP2 antibody titres of individual animals, with bars representing the mean and error bars representing standard deviation. Dotted lines represent designated cut-offs, above which signify anti-VP2 antibody-positive sera. The number of animals alive over the course of infection is designated above arrows at the top of each plot.

Whilst we have so far identified roles for specific T-cell subsets in antibody responses against BTV VP7 and NS2 proteins, it is not known whether these correlate to protection from re-infection with either homologous or heterologous BTV serotypes. Neutralising antibodies generated against the outer viral coat protein, VP2, confer protection against homologous BTV serotypes in infected ruminants. We next aimed to investigate whether T cells may facilitate or inhibit this protective neutralising antibody response. Selected time course sera from BTV-infected sheep of each treatment group were assessed by SNT to initially detect antibodies that completely neutralised the virus (i.e., fully protected cells and an absence of BTV infection in the cell monolayer). These antibodies with fully neutralising activity were detectable from 8 dpi to 20 dpi in the BTV-infected sheep, with high variation observed between individual animals (**Figure 9A**). On average, seroconversion was observed by 12 dpi in all mock, WC1^+^ γδ, and CD8^+^ T cell-depleted sheep surviving to 21 dpi, but was notably delayed to 14 dpi and 20 dpi in two of the three surviving CD4^+^ T cell-depleted sheep (**Figure 9A**). Whilst time to seroconversion was significantly different between depleted sheep (*p* = 0.014), the low number of sheep remaining within each treatment group at these later time points precluded identification of which groups differed. The neutralising antibody titres themselves were significantly different between depleted sheep at 12 dpi (*p* = 0.005), 14 dpi (*p* = 0.019), and 20 dpi (*p* = 0.039); however, no significant differences were identified between the T cell- and mock-depleted sheep (*p* > 0.05). It is likely that this lack of significance is hampered by the premature euthanasia of four of the seven CD4^+^ T cell-depleted sheep, as our data are based solely on the three surviving individuals, providing poor statistical power.

In addition to fully neutralising activity, our SNT also allowed for the detection of antibodies in serum which reduced virus infectivity but did not fully neutralise the virus (i.e., reduced viral infection in the cell monolayer compared to the positive control). Here, we defined partially protected cell monolayers as those with less than 20% BTV infection remaining in the cell monolayer compared to 50%–100% infection in the positive control. Antibodies with partial neutralising activity were detected earlier, from 7 dpi to 12 dpi (**Figure 9B**), than those with fully neutralising activity (8 dpi to 20 dpi). There was no significant difference in the time of detection of antibodies with partial neutralising activity between mock- and T cell-depleted sheep (*p* = 0.369) (**Figure 9B**). The titres of antibodies with partial neutralising activity were significantly different at seroconversion in mock-depleted sheep compared to the CD4^+^, CD8^+^, and WC1^+^ γδ T cell-depleted sheep (*p* = 0.043), with significantly lower titres observed in CD4^+^ T cell-depleted sheep (mean 45.39, range 40.00–56.57) compared to mock-depleted sheep (mean 185.5, range 50.40–508.00) (*p* = 0.016). Whilst antibody titres of partial neutralising activity were significantly different across treatment groups at 12 dpi (*p* = 0.044), 14 dpi (*p* = 0.001), and 20 dpi (*p* = 0.018), we were unable to elucidate which groups differed significantly, likely due to the low number of animals remaining within each treatment group at these later time points (*p* > 0.05).

## 4 Discussion

Orbiviruses are a major cause of ruminant morbidity and mortality worldwide and have demonstrated an ability to emerge in new regions outside of their recorded geographical range with severe economic and welfare consequences. In this study, we use a highly representative host–virus–vector transmission model to demonstrate the function and importance of CD4^+^, CD8^+^, and WC1^+^ γδ T cells in unprecedented detail during BTV infection of the natural host. Through selective depletion of specific T-cell subsets *in vivo* using mAbs, we were able to examine their impact on clinical disease outcomes, viral dynamics and clearance, serological and cellular immune responses, and pathogenesis in sheep as well as onwards transmission to the *Culicoides* vector. We reveal an underexplored role for T cells in facilitating timely protective immune responses and pathogenesis during BTV infection and highlight that CD4^+^ T cells may be particularly important targets of future vaccine design.

Depletion of circulating CD8^+^ T cells during BTV infection was associated with reduced disease severity, improved clinical outcomes, and earlier production of VP7-specific IgG antibodies, highlighting that this cytotoxic T-cell subset contributes to disease manifestation and immunopathological mechanisms. CD4^+^ T-cell depletion elicited the most substantial impact on host antibody responses, resulting in a likely impaired neutralising antibody response, earlier seroconversion of anti-VP7 antibodies, and abrogated or delayed production of anti-NS2 IgG antibodies. Depletion of both CD4^+^ and WC1^+^ γδ T cells also resulted in increased clinical scores and severity, indicating their potential importance for a timely protective immune response against BTV. Surprisingly, given their demonstrated role in response to BTV infection, depletion of T-cell subsets had no recorded impact on viral dynamics in the host (including the onset and peak of viraemia) or viral clearance post-infection. There was also no significant impact of T-cell subset depletion on the BTV infection rate in *Culicoides* fed on sheep at peak viraemia, which was instead driven by sheep viraemia levels. Whilst lymphocyte population dynamics were primarily influenced by viral replication, we found here that the presence of CD4^+^ and WC1^+^ γδ T cells appeared to be important for the characteristic expansion of CD8^+^ T cells after peak viraemia.

The generation of BTV-specific immunoglobulins varied considerably in response to infection, dependent upon the target antigen and/or presence of specific T-cell subsets. IgM-specific anti-VP7 immunoglobulins were generated in the absence of CD4^+^, CD8^+^, and WC1^+^ γδ T cells. However, whilst the class switching of anti-VP7 immunoglobulins occurred with comparable dynamics in the presence and absence of CD4^+^ T cells, class switching occurred significantly earlier in the absence of CD8^+^ T cells, suggesting that this subset impairs anti-VP7 IgG production. Our data also indicate, based on the timing of seroconversion, that the diagnostic BT cELISA likely detects, at least initially, IgM antibodies. The observed differences in seroconversion of anti-VP7 antibodies in the CD4^+^ T cell-depleted sheep between the two assays (cELISA and isotype-specific ELISA) are a likely reflection of differing assay sensitivity. In direct contrast to VP7, the production of IgM anti-NS2 antibodies during BTV infection was highly variable, with several sheep not seroconverting at all, whilst the majority of sheep that did seroconvert reached much lower comparable S/N antibody ratios when compared to VP7, regardless of T-cell depletion group. Interestingly, the production of IgG anti-NS2 antibodies in sheep surviving beyond 12 dpi was much more uniform. Only the depletion of CD4^+^ T cells substantially impacted the production of IgG anti-NS2 antibodies, with no anti-NS2 IgG detected in two of the surviving CD4^+^ T cell-depleted sheep and a notably delayed response in the third. This is particularly interesting given the otherwise comparable anti-NS2 IgG dynamics observed between CD8^+^ and WC1^+^ γδ T cell-depleted sheep and mock-depleted sheep. This is the first study to investigate antibody responses against a non-structural protein in BTV-infected ruminants, as previous studies have largely focused on the detection of protective neutralising anti-VP2 antibodies or antibodies against the immune dominant VP7 protein (27, 61). We highlight here the importance of studying humoral responses to a wide variety of BTV antigens to fully characterise the anti-BTV response repertoire in ruminants.

The neutralising capacity of anti-VP2 antibodies differed during BTV infection, initially involving only partially neutralising responses and followed later by partially and fully neutralising responses. Whilst we did not investigate isotype-specific anti-VP2 antibody responses in this study, it is inviting to speculate, based on the antibody subclass dynamics observed for other BTV proteins (VP7 and NS2), that the observed partial neutralising activity might be an IgM-dominated response, whilst full neutralisation might only be achieved in the presence of IgG-specific neutralising antibodies. Although not significant, the observed delay in seroconversion of neutralising anti-VP2 antibodies in the CD4^+^ T cell-depleted sheep and their significantly lower neutralising antibody titres at seroconversion might further highlight a difference in the T-cell dependency of different BTV antigens. Due to the increased clinical severity and premature euthanasia of several CD4^+^ T cell-depleted sheep, it is difficult to draw definitive conclusions, yet still tantalising to contemplate if BTV VP7 might be a T cell-independent antigen. VP7 has a highly repetitive, trimeric structure forming an icosahedral lattice on the outer core of the BTV particle (62, 63). Such rigid, highly repetitive antigenic structures are a characteristic feature of T cell-independent (type II) antigens, which efficiently class-switch in the absence of T cells through cross-linking B-cell immunoglobulin receptors (64–66). T cell-independent antigens also require the formation of intact antigen-specific germinal centres, which form in the absence of T cell-derived ligands such as CD40L (CD154) but require CD40 signalling, for example, provided through follicular dendritic cells (67). It has been reported that some BTV strains disrupt follicular dendritic cells and destroy germinal centres, thereby hindering B-cell division and resulting in a concordant delay in the production of affinity-matured, class-switched anti-VP7 and protective neutralising antibodies (17, 27–29). Hence, our findings in the present study of a gradual IgG anti-VP7 antibody response, yet rapid increase in the production of IgG anti-NS2 antibodies, might further point towards a difference in T-cell dependency between these two different viral antigens.

Here, we have shown that lymphocyte dynamics during BTV infection were driven by viral replication and not off-target effects from antibody-mediated depletion of specific T-cell subsets. We observed the characteristic pan-lymphopenia in sheep at peak viraemia but also found that the depletion of specific T-cell subsets influenced the dynamics of non-depleted lymphocyte populations. In investigating reciprocal influences between specific T- and B-cell populations, we identified a specific abrogation of the characteristic proliferative expansion of cytotoxic CD8^+^ T cells after peak viraemia in sheep (28, 29, 32) in the absence of CD4^+^ and WC1^+^ γδ T cells. This suggests that both CD4^+^ and WC1^+^ γδ T-cell subsets are important for this expansion and confirm the role of CD4^+^ T helper cells in activating and stimulating the proliferation of cytotoxic CD8^+^ T cells during viral infections (68). The timing of this CD8^+^ T-cell expansion in BTV-infected sheep differs from previous *in vivo* studies, where different viral strains were used, highlighting a potential link to virulence (28, 29, 32).

The data presented within this study also provide evidence that BTV suppresses serum IFN-γ and IL-4 cytokine production in infected sheep, as these cytokines were not detectable in any of the mock-depleted sheep during infection. This suppression of IFN-γ and IL-4 cytokine production appears to be abrogated by depletion of either of the three T-cell subsets but to different degrees. The onset of IFN-γ and IL-4 production from 6 dpi to 8 dpi and continuation thereafter demonstrated that these cytokines were still produced in response to BTV infection within the different T-cell subset-depleted sheep, rather than a response to the T-cell depletion itself. Effector functions of activated, mature CD4^+^ T cells are dependent upon downstream cytokine signalling cascades, which polarise the immune response towards a Th1 pro-inflammatory anti-viral response or Th2 anti-inflammatory defence (68, 69). Th1 and Th2 cytokines can also be produced by a range of immune cells including CD8^+^ and WC1^+^ γδ T cells and monocytes (28, 70, 71); however, previous *in vivo* infection studies in sheep report high variability in the detection of BTV-induced cytokines, dependent upon the strain of virus used for infection and associated virulence (29). We found that both representative Th1 (IFN-γ) and Th2 (IL-4) cytokines were not detected in sheep serum during BTV-4 infection. Whilst serum IFN-γ production has also previously been found to not be stimulated during BTV-1 or BTV-8 infection in sheep, IL-4 was shown to be significantly increased at the later stages of infection (>9 dpi) in sheep infected with BTV-1 and BTV-8 (29). IL-4 is an immunoregulatory cytokine, and IFN-γ is a fundamental anti-viral cytokine critical for mounting innate and adaptive immune responses (e.g., macrophage/cytotoxic T-cell activation, enhanced phagocytic activity, and MHC class II antigen presentation) (25, 72). It is not known whether the changes observed in serum cytokine dynamics in the presence and absence of different T-cell subsets were a direct result of impairment of T-cell effector function or if T-cell depletion resulted in an altered cytokine milieu due to indirect effects on other immune cell populations. Whilst serum cytokine dynamics are informative and provide oversight, they cannot determine which cells are responsible for the observed cytokine production. Future functional T-cell studies would now be of interest to elucidate whether this altered cytokine milieu is a result of production by specific T-cell subsets or whether other immune cell populations, likely innate immune cell lineages such as natural killer cells, may be stimulated upon T-cell subset depletion. As we found no clear trend evident either between the production of these serum cytokines and disease manifestation or viral clearance in sheep across the depletion groups, we believe our combined findings point towards a BTV-induced cytokine milieu, which may be strain-specific and may relate to the virulence characteristics of the virus (29, 70), although a functional role for specific cytokines in either immune protection or immunopathogenic mechanisms remains to be identified.

Whilst T cells did not directly impact BTV infection dynamics, we did observe a dose dependency of viraemia based upon the number of infected *Culicoides* taking a blood meal from sheep, but only in the absence of WC1^+^ γδ T cells. Non-conventional WC1^+^ γδ T cells form a major immune population within ruminant peripheral blood and are known to have wide-ranging innate and adaptive immune functions including protective immunity, IFN-γ signalling, cytotoxic activity (through perforin expression), and immune surveillance through migration between peripheral blood and local skin tissues (71, 73, 74). Whilst WC1^+^ γδ T cells can become productively infected in the skin during BTV infection (31) and influx into the site of *Culicoides* blood-feeding (75), it has yet to be determined whether they have a specific role at the local skin biting site during BTV infection. Our data provide further evidence that WC1^+^ γδ T cells may be important for BTV infection within the local skin environment and subsequent dissemination. Upon depletion of WC1^+^ γδ T cells, BTV may be less efficiently drained to regional lymph nodes and then general circulation, which could provide an explanation of the dose-dependent viraemia onset observed in their absence. Despite the depletion of WC1^+^ γδ T cells being associated with poorer clinical outcomes, this subset did not have a role in neutralising or non-neutralising anti-BTV antibody-mediated protection. Depletion of this regulatory T-cell subset previously increased IgM- and IgA-specific anti-viral antibody responses during respiratory syncytial virus infection in cattle (76). Here, we highlight for the first time a likely role for WC1^+^ γδ T cells in the expansion of the CD8^+^ T-cell population during BTV infection in sheep.

In this comprehensive *in vivo* infection study, we were able to efficiently deplete circulating CD4^+^, CD8^+^, and WC1^+^ γδ T-cell subsets in sheep to investigate their role during BTV infection directly within its natural host. Previously, mAbs have been used to deplete individual T-cell subsets in ruminants to determine their function in viral pathogenesis (36, 77, 78). Whilst we did not confirm whether T cells were efficiently depleted from lymphatic organs in this study, several *in vivo* T-cell depletion studies have previously shown equivalent concentrations (2.00–2.58 mg/kg) of anti-CD4/CD8 mAbs to be sufficient to achieve substantial depletion within the lymphatic organs (lymph nodes, spleen, and Peyer’s patches) as well as the blood of small and large ruminants, despite their gradual return to the circulation in similar dynamics to those we observed here (36, 76, 79). The already complex *in vivo* study and regular sampling schedule made additional invasive sampling procedures unfeasible, and it was deemed ethically unwarranted to utilise additional animals to confirm previous findings from the literature.

Our study has highlighted a likely important role for CD4^+^ T cells in protective, anti-viral immunity through the provision of a timely, rapid, protective neutralising antibody response and for the production of IgG anti-NS2 antibodies. The absence of CD8^+^ T cells was, in contrast, found to be associated with improved clinical outcomes, likely a result of reduced immune-mediated damage, but were also found to result in earlier production of anti-VP7 IgG antibodies, demonstrating that it is still unknown if anti-VP7 antibodies are of any consequence for BTV protection and viral clearance. We identified substantial differences in host antibody responses to different BTV proteins and hypothesised that the highly immune-dominant VP7 protein could be a T cell-independent antigen. WC1^+^ γδ T cells were also found to be likely important for reducing the clinical severity of BT disease. Whilst it is acknowledged that the observed effect size differences of some immunological and clinical parameters between depletion groups are small, most likely at least partly due to the observed early onset of substantial clinical disease resulting in several animals reaching humane clinical endpoints, these data present new insights into the role of T-cell subsets during BTV infection in its natural sheep host for the very first time and highlight several important avenues for future work in BTV immunology.

## Data availability statement

The original contributions presented in the study are included in the article/**Supplementary Material**. Further inquiries can be directed to the corresponding author.

## Ethics statement

The animal study was approved by The Animal Welfare and Ethics Review Board at The Pirbright Institute. The study was conducted in accordance with the local legislation and institutional requirements.

## Author contributions

KN: Conceptualization, Data curation, Formal analysis, Investigation, Methodology, Project administration, Supervision, Visualization, Writing – original draft, Writing – review & editing. NK: Investigation, Writing – review & editing. AF: Investigation, Writing – review & editing. KC: Investigation, Writing – review & editing. SG: Data curation, Formal analysis, Funding acquisition, Methodology, Visualization, Writing – review & editing. WD: Resources, Writing – review & editing. CS: Investigation, Writing – review & editing. MB: Investigation, Writing – review & editing. LC: Investigation, Writing – review & editing. AC: Investigation, Writing – review & editing. MA: Investigation, Writing – review & editing. JF: Resources, Writing – review & editing. CB: Resources, Writing – review & editing. JS: Investigation, Writing – review & editing. BS-B: Investigation, Writing – review & editing. SC: Conceptualization, Funding acquisition, Supervision, Writing – review & editing. KM: Conceptualization, Funding acquisition, Methodology, Resources, Supervision, Writing – review & editing. KD: Conceptualization, Funding acquisition, Investigation, Methodology, Project administration, Supervision, Writing – review & editing.
