# Supplementary material for: Specific T-cell subsets have a role in anti-viral immunity and pathogenesis but not viral dynamics or onwards vector transmission of an important livestock arbovirus
Source: Front Immunol. 2024 Jan 31;15:1328820. doi: 10.3389/fimmu.2024.1328820 (PMC10864546; doi:10.3389/fimmu.2024.1328820)
Supplement: Supplementary file 15 [file DataSheet_15.docx]

Supplementary Material

# Supplementary Data

**Data S1.** Raw data summarising immune cell population dynamics in whole blood of T cell/mock depleted sheep of experimental replicate 1 during BTV infection as assessed by immunolabelling and multicolour flow cytometry.

**Data S2.** Raw data summarising immune cell population dynamics in whole blood of T cell/mock depleted sheep of experimental replicate 2 during BTV infection as assessed by immunolabelling and multicolour flow cytometry.

**Data S3.** Raw data summarising immune cell population dynamics in whole blood of T cell/mock depleted sheep of experimental replicate 3 during BTV infection as assessed by immunolabelling and multicolour flow cytometry.

**Data S4.** Raw data summarising immune cell population dynamics in whole blood of T cell/mock depleted sheep of experimental replicate 4 during BTV infection as assessed by immunolabelling and multicolour flow cytometry.

**Data S5.** Raw data summarising immune cell population dynamics in whole blood of T cell/mock depleted sheep of experimental replicate 5 during BTV infection as assessed by immunolabelling and multicolour flow cytometry.

**Data S6.** Absolute numbers of live cells, single cells, lymphocytes/monocytes, CD4^+^, CD8^+^ or WC1^+^ γδ T cells and CD21^+^ B cells collected per whole blood sample from a representative mock depleted sheep across each experimental replicate as assessed by immunolabelling and multicolour flow cytometry.

**Data S7.** Raw data summarising the number of sheep alive per day, cumulative clinical scores, severity index and time of peak clinical score or peak clinical scores themselves in T cell/mock depleted sheep throughout BTV infection.

**Data S8.** Raw data summarising Cq values and BTV genome copies per ml EDTA blood detectable in T cell/mock depleted sheep throughout BTV infection by qRT-PCR.

**Data S9.** Raw data summarising the number of blood-fed *Culicoides* midges with a transmissible infection (Cq < 25) as determined by BTV Segment 10 qRT-PCR following feeding on T cell/mock depleted sheep to establish infection.

**Data S10.** Raw data summarising Cq values (as determined by BTV Segment 10 qRT-PCR) of pooled nasal and ocular swabs samples taken from T cell/mock depleted sheep at various time points during peak viremia.

**Data S11.** Raw data summarising Cq values and BTV genome copies detectable in *Culicoides* midges blood-fed on T cell/mock depleted sheep at peak viremia (day indicated) to investigate whether T cells have a role in onwards transmission of BTV to the insect vector.

**Data S12.** Raw data summarising the quantities of IFN-γ and IL-4 cytokines in the serum of T cell/mock depleted sheep during infection with BTV-4 MOR2009/07 as determined by quantitative sandwich ELISA.

**Data S13.** Raw data summarising dynamics of anti-BTV antibodies detectable in the serum of T cell/mock depleted sheep throughout BTV infection. OD Ratios of IgM- and IgG-specific anti-VP7 and anti-NS2 antibodies were determined by isotype BTV protein ELISAs, neutralising anti-VP2 antibodies (with fully or partially and fully neutralising activity) by SNT and S/N% of anti-VP7 antibodies by cELISA.

**Data S14.** Raw data summarising comparative OD ratios and quantitative titres of IgM and IgG specific anti-VP7 antibodies detectable in the serum of selected T cell/mock depleted sheep during BTV infection.

# Supplementary Figures and Tables

## Figures

**
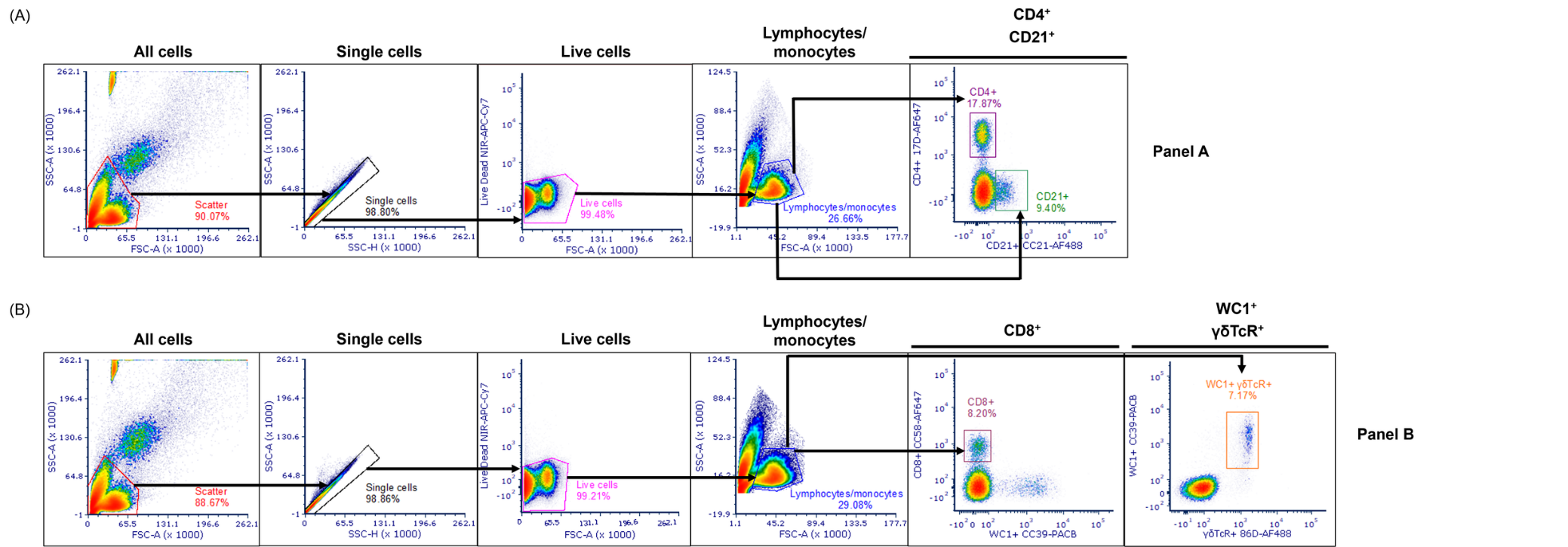
**

**Figure S1**. Gating strategy used for immunophenotyping whole blood using multicolour flow cytometry. Representative flow plots illustrating the gating strategy used to assess CD4^+^, CD8^+^ and WC1^+^/γδ TcR^+^ T cell and CD21^+^ B cell labelling of ovine whole blood, each gated within the live, singlet lymphocyte/monocyte cell population using multicolour flow cytometry. Forward and side scatter channels were initially used to identify the singlet lymphocyte/monocyte population, with a live-dead near-infrared dye included to enable gating on the live cell population. Whole blood was labelled with specific monoclonal antibodies from **(A)** panel A to assess CD4^+^ T cell and CD21^+^ B cell populations and **(B)** panel B to assess CD8^+^ and WC1^+^/γδ TcR^+^ T cell populations.

**
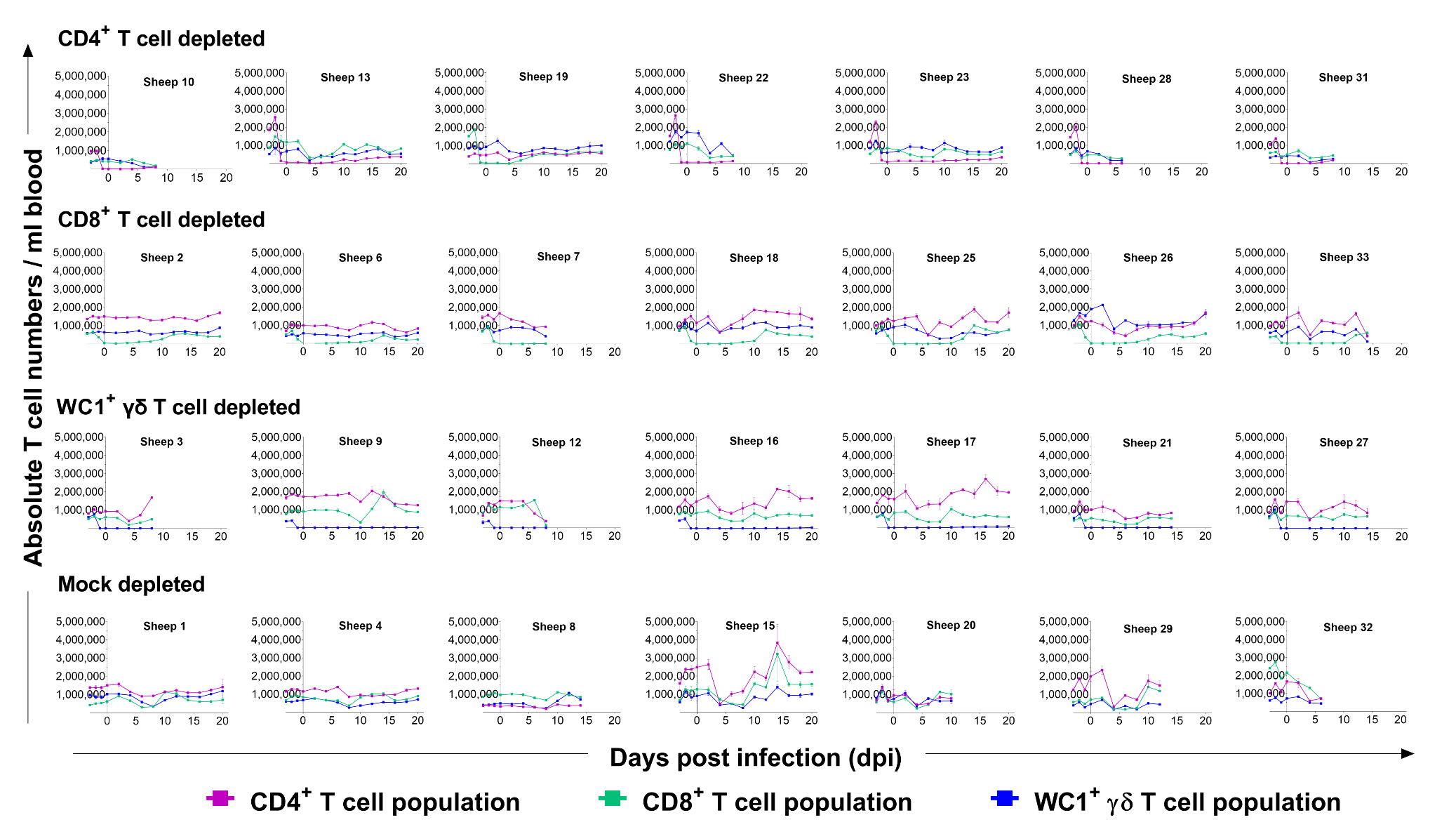
**

**Figure S2.** Absolute CD4^+^, CD8^+^ and WC1^+^/γδ TcR^+^ T cell numbers in T cell or mock depleted sheep during BTV infection. Mean (± SD) absolute numbers of CD4^+^ (purple), CD8^+^ (green) or WC1^+^/γδ TcR^+^ (blue) T cells detected per ml blood in individual T cell or mock depleted sheep during BTV infection by multicolour flow cytometry. Mean values represent triplicate technical replicates for each individual sheep.


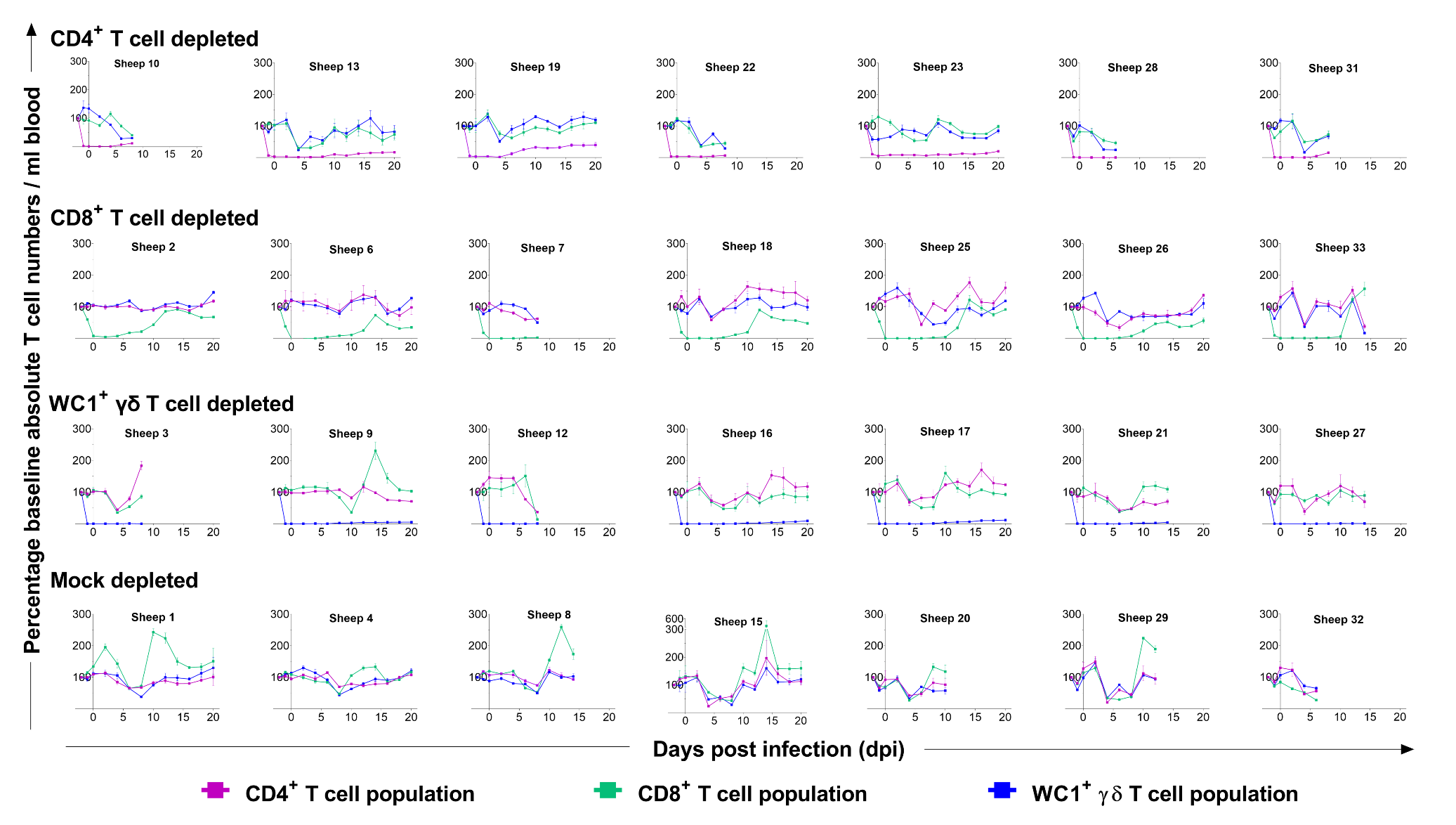


**Figure S3.** Percentage of baseline absolute CD4^+^, CD8^+^ and WC1^+^/γδ TcR^+^ T cell numbers in T cell or mock depleted sheep during BTV infection. Mean (± SD) percentage of baseline (pre-depletion) absolute CD4^+^ (purple), CD8^+^ (green) and WC1^+^/γδ TcR^+^ (blue) T cells per ml blood detected in T cell or mock depleted sheep during BTV infection by multicolour flow cytometry. Percentage of baseline absolute T cell numbers per ml blood at each day post infection (dpi) = pre-depletion (-3/-2 dpi) absolute cell number per ml blood / post-depletion absolute cell number per ml blood x 100. Mean values represent triplicate technical replicates for each individual sheep.

**
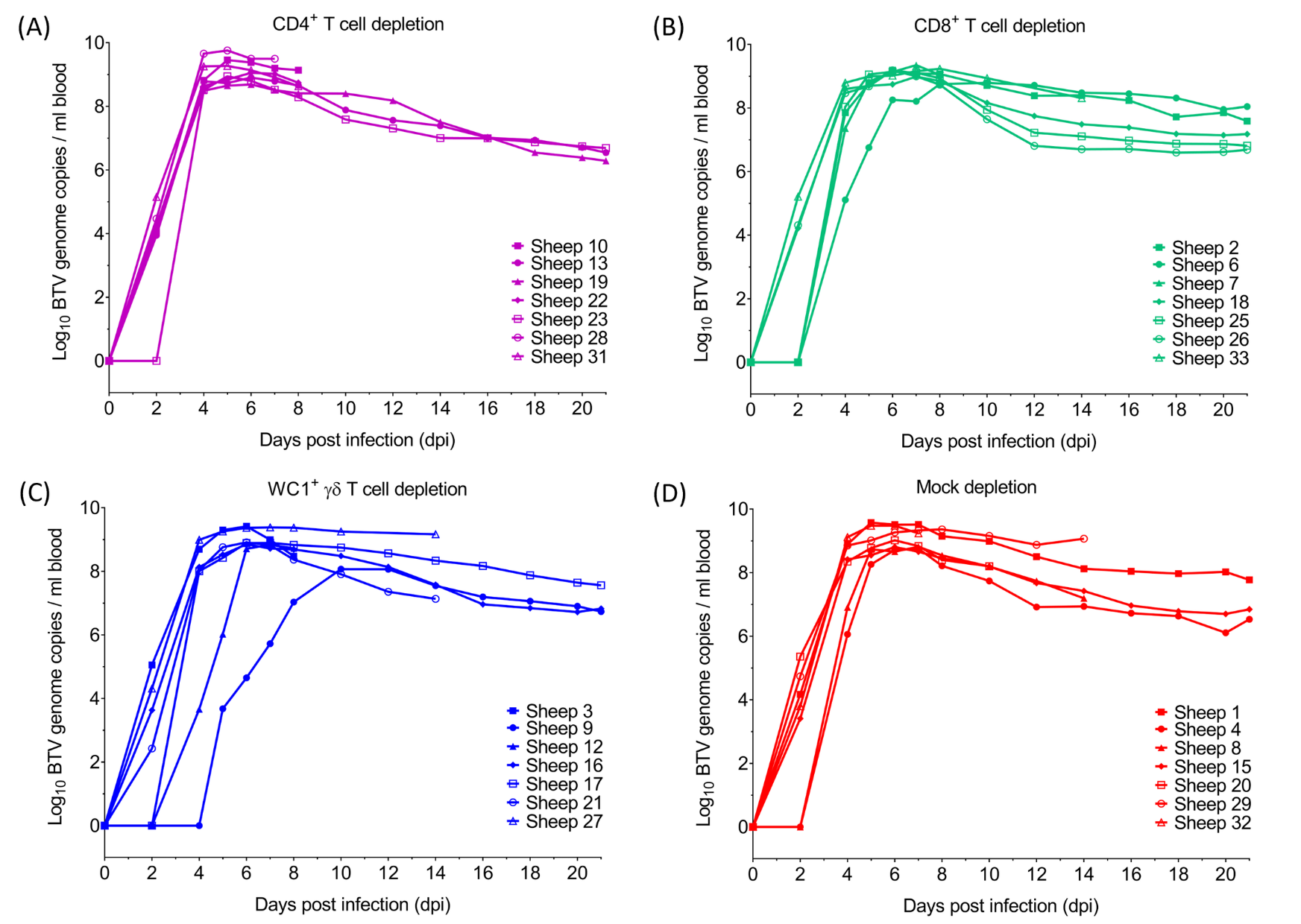
**

**Figure S4.** Viremia dynamics in individual T cell or mock depleted sheep during infection with BTV-4 MOR2009/07. Log_10_ BTV genome copies detected per ml blood in individual sheep from 0 to 21 days post infection (dpi) with BTV-4 MOR2009/07 following depletion of **(A)** CD4^+^ (n=7), **(B)** CD8^+^ (n=7) or **(C)** WC1^+^ γδ (n=7) T cells or **(D)** mock depletion (n=7) to investigate the role of individual T cell subsets on viremia dynamics.

**
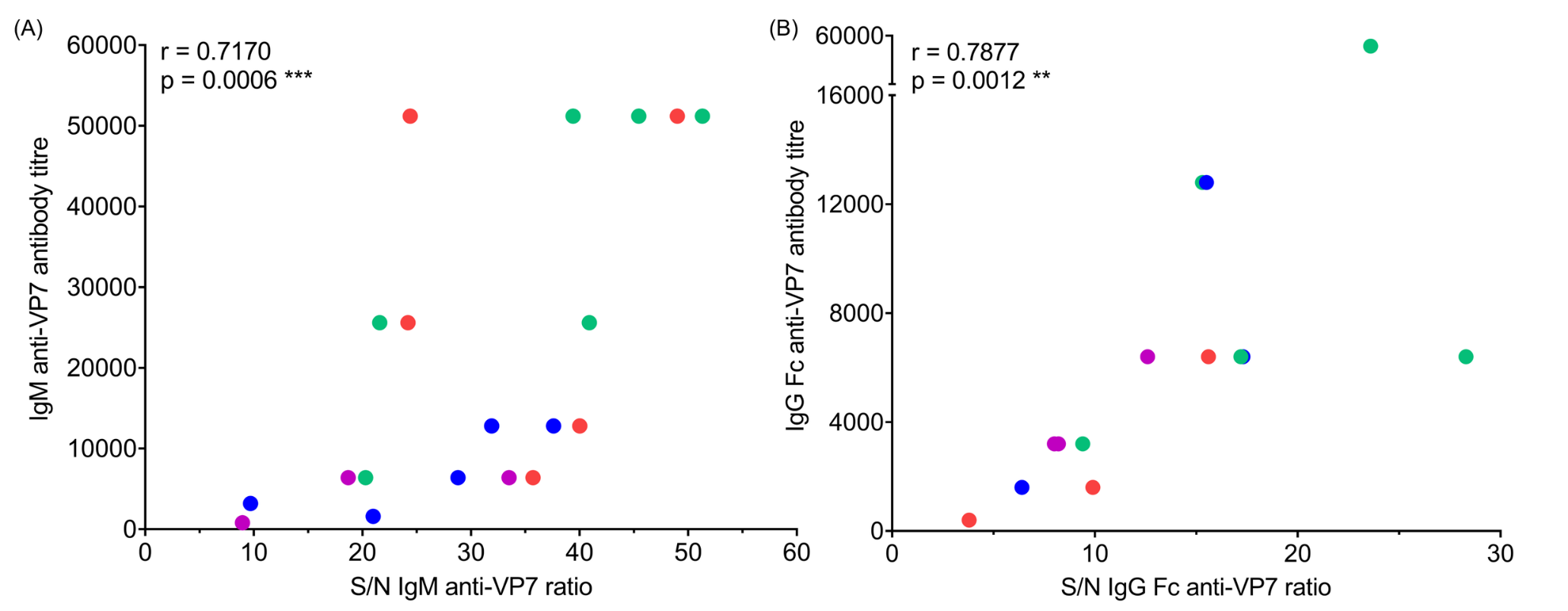
**

**Figure S5.** S/N ratios of anti-VP7 IgM and IgG antibodies show strong positive correlation to quantitative titre. Anti-VP7 **(A)** IgM and **(B)** IgG antibodies were detected in BTV-infected sheep depleted of CD4^+^ (purple), CD8^+^ (green) or WC1^+^ γδ (blue) T cells or mock depleted (red) using subclass-specific BTV VP7 protein ELISAs. Selected post-infection sera with both low and high sample / negative (S/N) antibody ratios for anti-VP7 IgM and IgG antibodies across each depletion group (n=3 or more per group) were titrated across a 2-fold dilution series (1:100 to 1:204,800) by ELISA (alongside matched pre-infection sera; titrated 2-fold from 1:100 to 1:800) to determine quantitative antibody titres which were expressed as the reciprocal of the highest dilution at which post-infection sera OD values exceeded those of matched pre-infection sera at the 1:100 dilution. These antibody titres were then correlated back to the S/N antibody ratios to determine whether they were representative. Anti-VP7 IgM and IgG antibody S/N ratios demonstrated a significant (*p* = 0.001 and 0.001) strong positive correlation to quantitative antibody titres (correlation coefficient, r = 0.717 and 0.788), demonstrating OD ratios are highly representative of the antibody quantities present in sheep sera.

## Tables

**Table S1. Mean rectal temperatures of T cell and mock depleted sheep during infection with BTV-4 MOR2009/07.** Mean rectal temperatures (°C) of CD4^+^ (n=7), CD8^+^ (n=7), WC1^+^ γδ (n=7) T cell and mock (n=7) depleted sheep and uninfected, non-depleted transmission control (n=5) sheep during infection with BTV-4 MOR2009/07.

|  | **Mean rectal temperature (°C)** | | | | |
| --- | --- | --- | --- | --- | --- |
| **Days post infection** | **CD4** | **CD8** | **WC1** | **Mock** | **Control** |
| **-2** | 39.0 | 39.1 | 39.0 | 38.8 | 38.6 |
| **0** | 39.0 | 39.2 | 39.1 | 39.2 | 39.0 |
| **2** | 39.1 | 39.2 | 39.1 | 39.1 | 39.1 |
| **4** | 39.8 | 39.4 | 39.1 | 39.3 | 38.6 |
| **5** | 40.0 | 39.7 | 39.6 | 39.4 | 38.8 |
| **6** | 40.2 | 39.7 | 39.9 | 39.7 | 38.8 |
| **7** | 40.0 | 40.0 | 40.4 | 40.7 | 38.8 |
| **8** | 40.4 | 40.1 | 40.9 | 40.8 | 38.8 |
| **9** | 39.7 | 40.3 | 40.2 | 40.1 | 39.0 |
| **10** | 39.2 | 40.0 | 39.7 | 39.5 | 39.2 |
| **12** | 39.3 | 39.3 | 39.0 | 39.3 | 38.9 |
| **14** | 39.4 | 39.4 | 39.3 | 39.4 | 38.9 |
| **16** | 38.9 | 39.0 | 38.7 | 39.3 | 38.9 |
| **18** | 39.2 | 39.3 | 38.7 | 39.0 | 39.0 |
| **20** | 39.4 | 39.5 | 38.9 | 39.0 | 39.0 |

**Table S2. Median, range and statistical significance of peak clinical scores and time to peak clinical scores in T cell and mock depleted sheep during infection with BTV-4 MOR2009/07.** Time to peak clinical score is given as days post infection (dpi). Statistical significance determined using a non-parametric Kruskal-Wallis test and post-hoc Dunn’s multiple comparison with a *p* value less than 0.05 considered statistically significantly.

|  | **Peak clinical score** | | | | **Time to peak clinical score (dpi)** | | | |
| --- | --- | --- | --- | --- | --- | --- | --- | --- |
|  | **Kruskal-Wallis** | | | **Dunn’s** | **Kruskal-Wallis** | | | **Dunn’s** |
| **Depletion group** | **Median** | **Range** | ***p-*value** | **Significance to mock**  **(*p*-value)** | **Median** | **Range** | ***p*-value** | **Significance to mock**  **(*p*-value)** |
| **CD4** | 9.0 | 4.0-12.5 | ns^^^  0.785 | ns^^^  (> 0.999) | 8.0 | 6.0-11.0 | Yes  0.010 | ns^^#^  (> 0.999) |
| **CD8** | 7.0 | 3.0-11.5 |  | ns^^^  (> 0.999) | 9.0 | 8.0-20.0 |  | Yes^#^  (0.025) |
| **WC1** | 8.0 | 2.5-12.0 |  | ns^^^  (> 0.999) | 8.0 | 8.0-11.0 |  | ns^^^  (0.656) |
| **Mock** | 7.8 | 5-13 |  | - | 8.0 | 7.0-9.0 |  | - |

**Table S3. Nasal and ocular shedding of BTV RNA in T cell and mock depleted sheep infected with BTV-4 MOR2009/07.** Segment 10 qRT-PCR was used to detect presence of BTV RNA (defined by Cq value) in nasal and ocular swab pools taken at 4-, 6-, 8-, 10- and 12-days post infection in CD4^+^, CD8^+^, WC1^+^ γδ T cell or mock depleted sheep. Mean and range Cq values given where applicable.

|  | **Nasal swab pools** | | | | **Ocular swab pools** | | | |
| --- | --- | --- | --- | --- | --- | --- | --- | --- |
| **Depletion Group** | **PCR positive**  **(n)** | **PCR negative**  **(n)** | **Mean Cq** | **Range Cq** | **PCR positive**  **(n)** | **PCR negative**  **(n)** | **Mean Cq** | **Range Cq** |
| **CD4** | 6 | 1 | 34.16 | 28.59 – 37.73 | 0 | 7 | - | - |
| **CD8** | 5 | 2 | 34.47 | 31.76 – 36.96 | 2 | 5 | 38.13 | 37.87 – 38.38 |
| **WC1** | 5 | 2 | 35.62 | 33.55 – 37.76 | 1 | 6 | 38.17 | - |
| **Mock** | 5 | 2 | 36.37 | 35.02 – 37.94 | 1 | 6 | 38.10 | - |
